# Linking gastrointestinal tract structure, function, and gene expression signatures to growth variability in broilers: a novel interpretation for flock uniformity

**DOI:** 10.1016/j.psj.2024.104158

**Published:** 2024-08-02

**Authors:** Muhammad Zeeshan Akram, Ester Arévalo Sureda, Matthias Corion, Luke Comer, Nadia Everaert

**Affiliations:** ⁎ Nutrition and Animal-Microbiota Ecosystems Laboratory, Department of Biosystems, KU Leuven, 3000-Heverlee, Belgium; † Precision Livestock and Nutrition Unit, Gembloux Agro-Bio Tech, University of Liège, Gembloux, Belgium

**Keywords:** flock uniformity, broiler production, transcriptomics, feed efficiency, growth rate

## Abstract

Variation in body weight (**BW**) within broiler flocks is a significant challenge in poultry production. Investigating differences in gut-related parameters between low (**LBW**) and high BW (**HBW**) chicks may provide insights into the underlying causes of BW heterogeneity. 908 day-old male broiler chicks were reared until d 7 and then ranked into LBW and HBW groups. Thereafter, performance parameters were compared between BW groups periodically. On d 7, 14, and 38, visceral organ characteristics, intestinal permeability, and duodenal and ileal histomorphology were examined. Expression profiles were analyzed for 79 ileal genes related to gut barrier function, immune function, nutrient transport, gut hormones, nutrient receptors, metabolism, and oxidation using high-throughput qPCR. Student's t-tests were performed to compare measurements. Multivariate statistics, including partial least square regression (**PLSR**) analysis, were applied to identify combinations of key genes discriminating BW groups, offering predictive capability for phenotypic variations. The HBW group remained heavier at each timepoint, which could be explained by higher feed intake. The HBW group had shorter relative small intestine length but higher villus height and villi height/crypt depth ratios. The LBW group demonstrated increased intestinal permeability on d 38. The LBW group showed upregulation of immune response genes including *TNF-α* on d 7 and *CYP450* on d 38, while the HBW group showed higher *AHSA1* and *HSPA4* expressions on d 7. The LBW group had upregulation of the metabolism genes *mTOR* and *EIF4EBP1* on d 7 and the satiety-induced hormone cholecystokinin on d 14, while the HBW group tended to increase expression of the hunger hormone ghrelin on d 38. Genes related to gut barrier function, nutrient transport, and oxidation categories were consistently upregulated in the HBW group. PLSR models revealed 4, 12, and 11 sets of key genes highly predictive of BW phenotypes on d 7, 14, and 38, respectively. These findings suggest that growth rates are linked to the intestinal size, structure, and function of broiler chickens, offering insights into the underlying mechanisms regulating BW.

## INTRODUCTION

Intensive genetic selection has significantly improved the production traits in modern broilers (Chang et al., 2016). Despite these efforts, significant body weight (**BW**) variation persists, leading to the grouping of flocks into two extreme BW categories, low (**LBW**) and high (**HBW**) BW broilers (Akram et al., 2024). Higher BW variation is linked to compromised feed efficiency, increased mortality rates, and suboptimal growth trajectories within broiler flocks (Jacobs, 2016; Vasdal et al., 2019), raising concerns regarding uniformity in abattoir processing and potential economic losses (Hughes et al., 2017). This variability in growth performance seems to extend beyond conventional selection criteria and is influenced by multifaceted factors, including gut health. The gastrointestinal tract (**GIT**) in birds is a central organ for growth, which typically undergoes rapid development in the early stage of life to meet nutritional and immunological needs (Cardeal et al., 2020). Disparities in gut structure and function between LBW and HBW broilers could significantly impact production efficiency, even under standardized rearing conditions.

One of the mechanisms that potentially influences growth variations in LBW and HBW chickens could be the efficiency of nutrient uptake within the small intestine. The small intestine's significance for nutrient digestion and absorption in chickens is widely recognized (Uni et al., 1998). An optimally developed GIT with efficient histomorphological characteristics is pivotal for shaping long-term growth, metabolism, and overall health (El Sabry and Yalcin, 2023). Nutrient uptake is mediated by transporter proteins located at the brush border or basolateral membranes of the intestinal epithelia, governing the flux of nutrients from the intestinal lumen to the bloodstream (Kaminski and Wong, 2018). Among these transporters, amino acid, peptide, and monosaccharide transporters belong to the solute carrier (**SLC**) gene family, comprising 395 transporter genes across 52 families (Hediger et al., 2013). The regulation and expression of these specific nutrient transporters profoundly influence animal growth and development (Mott et al., 2008), with increased mRNA expression of glucose transporters previously associated with higher BW in chickens (Zhang et al., 2013).

Intestinal permeability is another gut-related mechanism believed to be capable of potentially influencing growth variation, given its critical role in integrity of the intestinal barrier regulated by tight junction (**TJ**) proteins (Gilani et al., 2016). In addition to TJ proteins, mucin also contributes significantly to this complex defense mechanism by serving as a physical barrier against harmful pathogens and toxins (Santos et al., 2024). Increased permeability can result in bacterial translocation and the entrance of toxin compounds into the body, affecting nutrient absorption and growth efficiency (Awad et al., 2017). Furthermore, the gut microbiome directly affects the development and function of the mucosal immune system (Kogut et al., 2020). Differences in microbiota between chickens of extreme BWs have been reported (Abdel-Kafy et al., 2022), with LBW chickens often showing a higher abundance of potential pathogens (Akram et al., 2024), which can trigger differential immune responses in birds differing in weight. Host-pathogen interaction in chickens can lead to shifts in energy distribution, potentially prioritizing immunity over rapid growth, impacting growth rates and potentially contributing to differences in broiler BW as observed in practice.

The next conceivable mechanism for growth variation concerns the regulation of appetite, which plays a crucial role in animal growth (Buzala and Janicki, 2016), given the direct relationship between feed intake and economic traits in broilers (te Pas et al., 2020). Gut hormones significantly modulate the feed intake in chickens, thereby influencing weight gain (Ceron-Romero et al., 2021). Feed preferences are established early in the life of birds (te Pas et al., 2020), and differences in feed intake from the outset can elicit diverse responses in chickens and directly influence growth homogeneity. Understanding the role of mediators affecting feed intake in broilers is essential due to its involvement in major physiological processes like growth, immunity, and production.

GIT size, structure, and function along with intestinal gene expression profiles have been identified as potential mechanisms in controlling the growth rate of chickens with different genotypes (Li et al., 2013; Metzler-Zebeli et al., 2018; Kinstler et al., 2024; Santos et al., 2024). However, our understanding of GIT development and intestinal gene expression patterns contributing to intra-flock variance among broilers under uniform management practices remains limited. Moreover, existing studies often focus on single time points, typically at slaughter age, overlooking crucial changes in intestinal physiological functions during earlier life stages that influence later growth trajectories. In this study, we investigated differences in visceral organ size, gut permeability, small intestine morphology and gene expression profiles related to gut barrier function, immune responses, nutrient receptors and transporters, neuropeptide gut hormones, metabolism, and oxidation between LBW and HBW broilers across different growth stages. Our objective was to elucidate mechanisms driving growth variations between LBW and HBW broilers reared under uniform management conditions on d 7, 14, and 38.

## MATERIALS AND METHODS

This animal study was approved by the Katholieke Universiteit Leuven Ethical Committee for Animal Experimentation (Ethical protocol P045/2022, Belgium) and was performed at TRANSfarm, the research facility for animal experimentation of KU Leuven (Lovenjoel, Belgium).

### Broiler management

A total of 908 day-old male ROSS 308 broiler chicks were obtained from Belgabroed NV (Merksplats, Belgium) and housed in separate floor pens (1.3 m^2^ each) at the research facility. The floor of the pens was cobvered with a 3 cm layer of wood shavings, serving as bedding material. The initial barn temperature was set at 33°C and was systematically lowered by approximately 0.5°C each day until reaching 21.5°C on the 21st d, after which it was held constant at that level. A 1-h dark period was initially provided until d 7, after which it was extended to 6 h for the remainder of the study period. The 38-d experiment ensured uniform rearing conditions and ad libitum access to the same feed and water for all chicks. The chicks were vaccinated against Newcastle disease and Gumboro on d 16 and had no exposure to antibiotics. Chicks were fed crumbled, sieved pellets as starter feed (1–14 d), transitioning to pelleted grower feed (15–28 d), and then to finisher feed (29-38 d, Supplementary file 1, Table S1).

### Experimental Design

The chicks were weighed on d 7, and they were categorized into three groups based on their BW: low body weight (**LBW**), middle body weight, and high body weight (**HBW**). LBW chicks (n = 294, 32%) were those with weights below the mean BW by half the standard deviation (½ × SD), while HBW chicks (n = 280, 31%) exceeded the mean BW by ½ × SD. Middle BW chicks (n = 334, 37%), falling within the mean BW ± ½ × SD, were excluded from the study. The experimental setup ensured uniform conditions for all birds, with 28 pens utilized in total (14 replicate pens per group) allocated for LBW (21 chicks/pen) and HBW (20 chicks/pen) groups. The LBW pens accommodated one additional bird to maintain a similar stocking density (kg/m^2^) to that of the HBW pens.

### Growth Performance

Individual bird weights and feed consumption per pen were recorded on d 7, 14, 28, and 38. Mortality was noted as it occurred. Subsequently, average daily gain (**ADG**), mortality-corrected average daily feed intake (**ADFI**), and feed conversion ratio (**FCR**) were calculated.

### Sampling and Visceral Organ Measurements

On d 7, 14, and 38 post-hatch, 20 birds per experimental group were sacrificed for sampling by electronarcosis followed by decapitation. Following the killing, organ dissection included the stomach (proventriculus and gizzard), small intestine, liver, pancreas, spleen, bursa of Fabricius, and heart. The small intestine weight was weighed without evacuating the digesta and its length recorded. Relative organ weights were calculated as grams per 100 grams of BW, and small intestine length as centimeters per 100 grams of BW. Duodenal and ileal segments (approximately 5 cm long from midpoint) were collected for histomorphological analysis. Ileal tissue samples were rapidly snap-frozen and stored at -80°C for subsequent target gene expression analysis. Fourteen chickens from each group were randomly chosen to evaluate intestinal permeability using fluorescein isothiocyanate dextran (FITC-dextran, Molecular weight 4,000 kda; Sigma-Aldrich, St. Louis, MO).

### Intestinal Histomorphology

Duodenum and ileum sections were rinsed with 1x phosphate-buffered saline followed by immersion in a 4% formaldehyde solution for 48 h for fixation, before preservation in 70% ethanol. Subsequently, the sections were embedded in paraffin and sectioned using a microtome. The resulting sections were then mounted on glass slides, stained with Alcian Blue-Periodic Acid Schiff, and examined under a microscope at 20x magnification. The selected sections were analyzed using NDP.view2 software (Hamamatsu Photonics K.K., Hamamatsu, Japan). For each sample, twenty well-oriented villus-crypt units were evaluated. The recorded parameters included villus height (**VH**), crypt depth (**CD**), VH:CD ratio, villus width, and the thickness of the submucosa and tunica muscularis layers.

### Intestinal Permeability

A solution containing FITC-dextran (2.2 mg/mL/bird) was administered via oral gavage, and blood samples (1 mL) were collected from the jugular vein 2.5 h postadministration. The obtained blood samples were centrifuged at 3000 × g for 15 min at 4°C to isolate the plasma. Diluted plasma samples and standard solution (1:5 PBS) were pipetted in duplicates into 96-well microplates, and fluorescence intensity measurements were then performed using spectrophotometry (Victor3, PerkinElmer Inc., Hopkinton, MA) with an excitation wavelength of 485 nm and an emission wavelength of 530 nm. The absolute FITC-dextran concentration was calculated based on the standard curve as ng/mL of blood. The relative concentration of FITC-dextran was calculated as ng/mL/100 g BW. Normalization of plasma FITC-dextran values to BW accounts for variations in BW and blood volume between BW groups. This allows for a more accurate comparison of intestinal permeability across chickens of different weights.

### RNA Extraction

RNA was extracted using the ReliaPrep RNA Miniprep Systems (Promega Corporation, Madison, WI) Kit as per the manufacturer's guidelines. RNA quantity and quality were determined via NanoDrop 2000 (Thermo Fisher Scientific, Waltham, MA), while integrity was confirmed using 1% agarose gel electrophoresis.

### Primer Design and Validation

The study investigated 92 genes related to various physiological functions of the ileum, selected from published literature. Exon-exon-spanning primers were either obtained from previous studies or designed using the NCBI Primer-Blast tool (Supplementary file 1, Table S2). These primers, under 30 nucleotides, produced amplicons not exceeding 150 base pairs. Efficiency and specificity of all primers were assessed on a QuantStudio 6 Real-Time PCR Systems (Thermo Fisher Scientific) using three-fold serial dilutions of a pool of cDNA from all samples. The validation of PCR products was carried out using agarose gel electrophoresis, which confirmed the presence of a single product, as well as through the analysis of melting curves during real-time PCR.

### Reverse Transcription and Preamplification

Reverse transcription of 50 ng of RNA was carried out using a Reverse Transcription Master Mix (Standard BioTools, South San Francisco, CA) as per the manufacturer's guidelines. A primer mix was made by pooling 1 µL each of forward and reverse primers with Tris EDTA buffer (Thermo Fisher Scientific) to a final volume of 400 µL. This primer mix was then combined with Fluidigm PreAmp Mastermix (Standard BioTools) to form a preamplification mix. In a 96-well PCR plate, 3.75 µL of preamplification mix was mixed with 1.25 µL of cDNA samples, and subjected to thermal cycling conditions: 2 min at 95°C, followed by 14 cycles of 95°C for 15 s and 60°C for 4 min. Exonuclease I (New England Biolabs, Ipswich, MA) was subsequently used to remove unincorporated primers. After treatment, samples were diluted ten-fold with Tris EDTA buffer and stored at –20°C.

### High-Throughput qPCR

Quantitative PCR (**qPCR**) was performed using the BioMark HD instrument with a 96.96 Dynamic Array integrated fluidic circuits (**IFC**) specifically designed for gene expression (Standard BioTools). A total of three IFCs were used, each dedicated to samples from different timepoints (d 7, 14, and 38), tested separately. A pool was created by combining 10 µL from each individual sample. Pre-amplified cDNA of the pooled samples was diluted threefold for primer efficiency and standard curve setup for each IFC. Non-template controls were included to monitor for contamination and nonspecific amplification. The sample mix comprised 0.25 µL 20X DNA Binding Dye (Standard BioTools) and 2.5 µL 2X SSoFast EvaGreen Supermix with low ROX (Biorad, Hercules). The assay mix contained 2.5 µL 2X Assay Loading Reagent (Standard BioTools) and 2.25 µL of 1x DNA Suspension Buffer (TEKnova, Hollister, CA). Thermal cycling in BioMark HD machine involved denaturation at 95°C for 60 s, followed by thirty cycles of denaturation at 96°C for 5 s, and annealing/elongation at 60°C for 20 s. The raw data of gene expression were extracted using SBI Real-Time PCR software (v1.0.2, Standard BioTools). Relative mRNA concentrations were determined using the standard curve of the pooled samples on each respective IFC. Housekeeping genes' expression stability according to the experimental groups and sampling time points was calculated using NormFinder (Andersen et al., 2004), and four housekeeping genes (TBP, B2M, NDUFA, and B-ACTIN) proved most stable over the groups and time points. The relative gene expression level for each target and housekeeping gene was calculated using the Pfaffl method (Pfaffl, 2001), and the geometrical mean of the relative expression of the 4 housekeeping genes (TBP, B2M, NDUFA, and B-ACTIN) was used to normalize all samples.

### Statistical Analysis

The data regarding growth performance, visceral organ characteristics, intestinal histomorphology, in vivo gut permeability, and ileum gene expression were analyzed via a linear mixed model in R (v4.2.3, R Foundation, Vienna, Austria). The BW effect was used as a fixed effect and the pen effect was considered a random effect to account for the possible confounding variation due to pen location and number of animals in each pen. Prior to analysis, normal distribution of the data was confirmed via Shapiro-Wilk's test. Subsequently, Student's *t*-test was applied to assess statistical significance, with *P* values < 0.05 indicating significance and values within 0.05 < *P* < 0.10 considered as trends. For gene expression data, P-values were adjusted for false discovery rate (**FDR**) using the Benjamini–Hochberg method (Yoav Benjamini and Yosef Hochberg, 1995), with a significance threshold set at < 0.05. Results are presented as means alongside a pooled SD, which combines the variability observed in all samples.

Principal component analysis (**PCA**), an unsupervised pattern recognition technique, was used in R using the factoextra package (v 1.0.7) to visualize overall patterns of gene expression data across BW groups. Genes were used as variables, with samples as individual data points, while BW was included as a categorical variable. Permutational multivariate analysis of variance (**PERMANOVA**) analysis was performed on PCA scores to assess whether there are significant differences between groups in terms of the overall multivariate structure captured by the principal components (**PC**). Heatmaps were generated in R using the pheatmap package (v 1.0.12) to visualize sample variability, with gene expression values scaled by row. Heatmaps were based on Pearson's correlation distance and ward clustering method for 2-way hierarchical clustering analysis.

Using gene expression datasets, partial least square regression (**PLSR**) models were built as an alternative to identify a combination of key genes predicting the growth rate of LBW and HBW broilers. Per day, nine cross-validation splits were created with two or three samples per BW group. The number of latent variables was determined for the highest R-squared of the cross-validation set (R^2^_CV_). The outlier analysis was performed by examining the Q residuals, the Hotelling T^2^, and manual inspection of aberrant gene expression levels in the data. Then, the PLSR model was further optimized by applying a variable importance in projection (**VIP**). Thereby, each variable was considered significant if its score was 1 or higher. The PLSR analysis was performed using the PLS toolbox (v8.7 2019, Eigenvector Research, Wenatchee, WA) within Matlab (v2018b, Mathworks, Natick, MA).

Pearson's correlation analysis was used to establish and quantify the relationship between BW and other performance parameters, intestinal size, structure, and function in R using the package corrplot (v0.92). Correlations with a *P* < 0.05 and |R| > 0.30 were reported.

## RESULTS

### Performance Parameters

**Figure 1:**
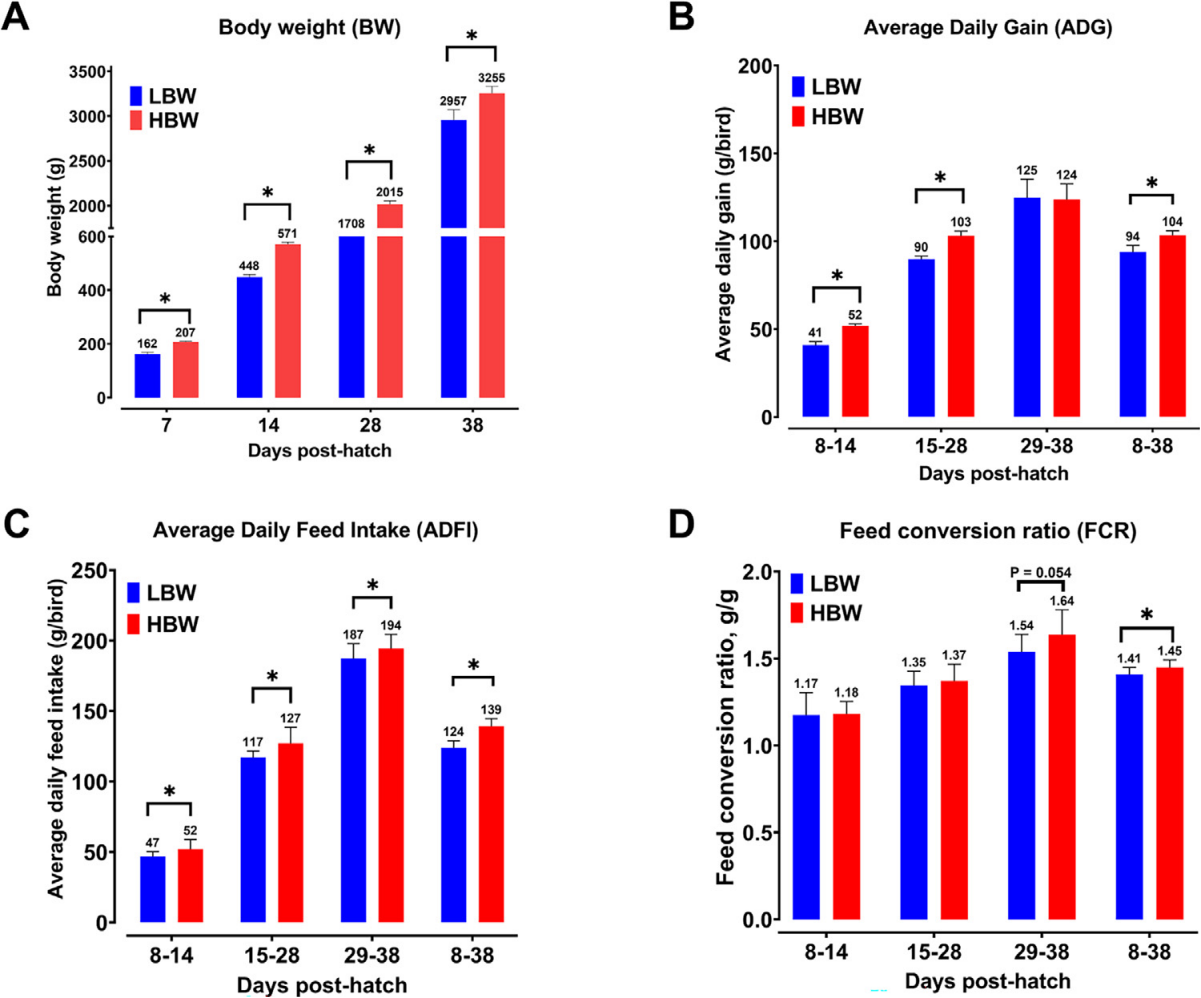
Body weight (A), average daily gain (B), average daily feed intake (C) and feed conversion ratio (D) of low (LBW, n = 14 pens) and high (HBW, n = 14 pens) body weight (**BW**) groups. Except for BW data, a pen was considered an experimental unit. BW was recorded from individual birds. Data are presented as mean and standard deviation (**SD**). Values with (*) indicate significant differences at *P* < 0.05 (Student's t-test).

The average BW of birds was 43.2 ± 2.88 g upon placement in the barn. On d 7, these chicks were divided into two distinct weight categories designated as LBW and HBW groups, showing a statistically significant difference (P < 0.05, Figure 1). Thereafter, the LBW group consistently maintained a lower BW (*P* < 0.05) on d 14, 28, and 38, when compared to their HBW counterparts. The ADG in LBW chicks demonstrated a significant reduction (*P* < 0.001) compared to HBW chicks during the periods of 7 to 14 d, 15 to 28 d, and the overall study duration of 7 to 38 d. However, the birds within the LBW group exhibited similar ADG during the 29-38-d period. Furthermore, LBW chicks exhibited lower ADFI (*P* < 0.05) than the HBW chicks throughout the study, while FCR was lower (*P* = 0.021) in the LBW group than in the HBW group during the overall period of 7 to 38 d.

### Visceral Organ Development

The chicks in the LBW group demonstrated higher relative heart weights on d 7 and 14 (*P* = 0.006 and *P* < 0.001, respectively, Table 1), as well as higher stomach and bursa relative weights on d 38 (P < 0.010). A tendency for increasing relative pancreas weight on d 14 was observed in LBW birds (*P* = 0.083). The LBW group demonstrated longer small intestine length on d 7, 14, and 38 (*P* < 0,001), despite lower relative weights of the small intestine on d 7 (*P* < 0.001) and 14 (*P* = 0.020).

**Table 1:** Visceral organ weights (g/100 g body weight) and small intestine length (cm/100 g body weight) of chickens from the low (**LBW**) and high (**HBW**) weight groups.

Items	Days	1Groups		SD	*P* value
		LBW	HBW		
Heart (g)	D 7	0.79^a^	0.70^b^	0.120	0.006
	D 14	0.85^a^	0.76^b^	0.082	<0.001
	D 38	0.49	0.49	0.066	0.679
Liver (g)	D 7	4.30	4.34	0.423	0.426
	D 14	3.42	3.26	0.400	0.192
	D 38	1.96	2.06	0.280	0.301
Spleen (g)	D 7	0.07	0.07	0.027	0.207
	D 14	0.08	0.09	0.018	0.633
	D 38	0.12	0.11	0.032	0.548
Pancreas (g)	D 7	0.44	0.42	0.087	0.591
	D 14	0.40	0.36	0.077	0.083
	D 38	0.17	0.15	0.040	0.168
Bursa (g)	D 7	0.19	0.16	0.056	0.882
	D 14	0.25	0.22	0.069	0.149
	D 38	0.17^a^	0.13^b^	0.044	<0.001
Stomach (g)	D 7	6.59	6.30	0.651	0.155
	D 14	4.75	4.38	0.535	0.033
	D 38	1.94	1.68	0.384	0.032
Small intestine (g)	D 7	16.80^a^	19.95^b^	1.393	<0.001
	D 14	15.20^a^	15.85^b^	1.596	0.020
	D 38	8.01	7.37	1.265	0.113
Small intestine (cm)	D 7	61.68^a^	50.11^b^	7.899	<0.001
	D 14	29.29^a^	26.57^b^	2.669	<0.001
	D 38	7.28^a^	6.21^b^	0.924	<0.001

1 LBW: low body weight group (n = 20), HBW: high body weight group (n = 20). The experimental unit was considered as individually sampled chickens. Data are presented as mean and pooled standard deviation (**SD**).

a-b Values with different superscripts in a row differ at *P* < 0.05 (Student's t-test).

### Duodenum and Ileum Histomorphology

The LBW group exhibited shorter duodenal VH on d 7 and 38 (*P* ≤ 0.001 and *P* ≤ 0.049, respectively), a lower VH:CD ratio, and a thinner tunica muscularis layer on d 7 compared to the HBW group (*P* < 0.05, Table 2). Birds in the HBW group demonstrated increased ileal VH (*P* ≤ 0.001 and *P* = 0.002, respectively) and greater VH:CD ratios on d 7 and 14 (*P* ≤ 0.001 and *P* = 0.013, respectively, Table 3).

**Table 2:** Duodenal histological characteristics of the chickens from low (**LBW**) and high (**HBW**) weight groups.

1Items	Days	2Groups		SD	P value
		LBW	HBW		
VH (μm)	D 7	1353^b^	1496^a^	120.7	≤0.001
	D 14	1885	1964	160.3	0.155
	D 38	2003^b^	2141^a^	218.6	0.049
CD (μm)	D 7	137	134	27.4	0.562
	D 14	199	212	40.7	0.381
	D 38	201	191	65.4	0.649
VH:CD	D 7	9.9^b^	11.9^a^	2.51	0.015
	D 14	9.71	9.70	2.10	0.962
	D 38	11.3	12.0	3.93	0.582
Villus Width (μm)	D 7	154	154	18.5	0.890
	D 14	178	186	26.1	0.246
	D 38	187	199	27.8	0.207
Sub mucosa (μm)	D 7	22.4	23.1	3.91	0.548
	D 14	21.8	22.9	3.62	0.330
	D 38	28.4	28.9	4.71	0.680
Tunica muscularis (μm)	D 7	127^b^	138^a^	16.7	0.042
	D 14	149	150	23.1	0.981
	D 38	199	186	33.5	0.348

1 VH: villus height, CD: crypt depth, VH:CD: ratio of VH to CD.

2 LBW: low body weight group (n = 20), HBW: High body weight group (n = 20); The experimental unit was considered as individually sampled chickens. Data are presented as mean and pooled standard deviation (SD).

a-b Values with different superscripts in a row differ at *P* < 0.05 (Student's t-test).

**Table 3:** Ileal histological characteristics of the chickens from low (**LBW**) and high (**HBW**) weight groups.

1Items	Days	2Groups		SD	*P* value
		LBW	HBW		
VH (μm)	D 7	504^b^	579^a^	67.1	≤0.001
	D 14	606^b^	688^a^	86.8	0.002
	D 38	1007	1087	147.7	0.089
CD (μm)	D 7	126	123	17.9	0.599
	D 14	188	174	28.8	0.133
	D 38	160	160	27.2	0.995
VH:CD	D 7	4.1^b^	4.8^a^	0.73	≤0.001
	D 14	3.3^b^	3.8^a^	0.70	0.013
	D 38	6.4	6.9	1.14	0.196
Villus Width (μm)	D 7	151	137	18.2	0.224
	D 14	179	182	15.6	0.539
	D 38	156	163	34.8	0.674
Sub mucosa (μm)	D 7	20.8	20.3	2.14	0.429
	D 14	26.9	28.2	3.86	0.315
	D 38	34.0	38.2	9.15	0.346
Tunica muscularis (μm)	D 7	111	116	21.7	0.615
	D 14	149	154	21.1	0.420
	D 38	191	223	53.9	0.197

1 VH: villus height, CD: crypt depth, VH:CD: ratio of VH to CD.

2 LBW: low body weight group (n = 20), HBW: High body weight group (n = 20); The experimental unit was considered as individually sampled chickens. Data are presented as mean and pooled standard deviation (SD).

a-b Values with different superscripts in a row differ at *P* < 0.05 (Student's t-test).

### Intestinal Permeability

**Figure 2:**
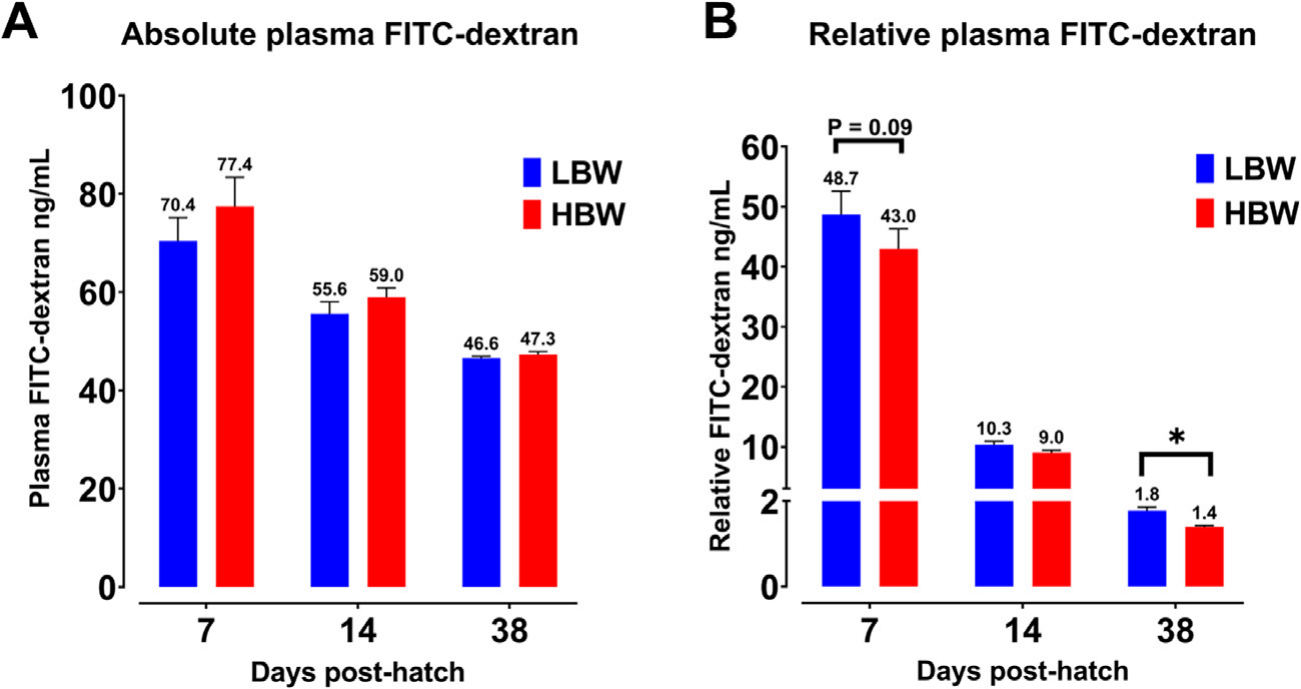
Plasma absolute (ng/mL; A) and relative (ng/mL/100 g body weight; B) fluorescein isothiocyanate dextran (FITC-dextran, 2.2 mg/mL/bird) levels of chickens from low (LBW, n = 14) and high (HBW, n = 14) body weight groups. Data are presented as mean and standard deviation (**SD**). Values with (*) significantly differ at *P* < 0.05 (Student's *t-*test).

Absolute plasma FITC-dextran levels on d 7, 14, and 38 did not differ between BW groups (Figure 2). When considering the plasma concentration of FITC-dextran relative to the BW of birds, the LBW group demonstrated a trend towards increased plasma FITC-dextran levels on d 7, and had significantly higher levels on d 38 compared to the HBW group (*P* ≤ 0.001).

### Ileum Gene Expression

One sample from the LBW group on d 7 and one sample from HBW on d 38 completely failed during high-throughput qPCR and were subsequently excluded from the study. Due to technical problems, *CLDN4, JAM 3, T1R1, TLR4, SLC5A9, FABP*, and *FABP1* on d 7, *IL-4, IL-10*, and *TLR4* on d 14, and *OCLN, IL-4, FABP*, and *FABP1* on d 38 were withdrawn from the study because of their low mRNA levels in all samples.

### Principal Component Analysis and Heatmap Clustering

**Figure 3:**
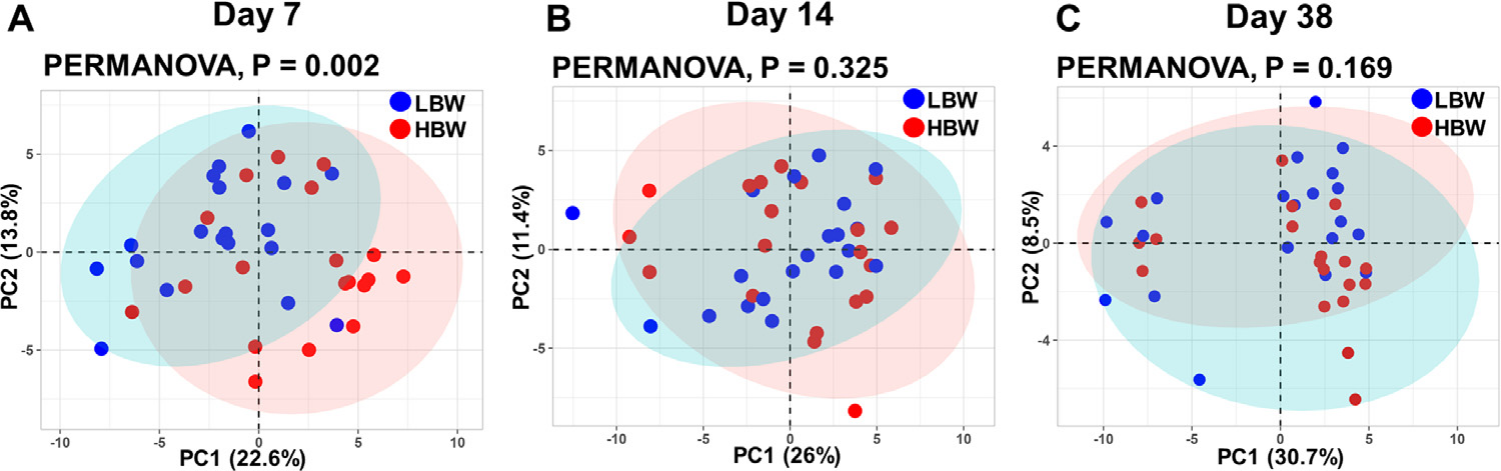
Principal component analysis (PC1 and PC2) based on the gene expression in the ileum of low (**LBW**) and high (**HB**W) body weight groups on d 7 (A), 14 (B), and 38 (C).

The PCA on d 7 showed a distinct clustering of samples based on their BW groups, with LBW and HBW samples separated along the PC1 axis (Figure 3A). This observation was further validated by the PERMANOVA analysis, which confirmed the substantial differences in the gene expression data represented by PCs, was significantly associated with the BW groups (*P* = 0.002). In contrast, on d 14 and 38, we still observed variation in the gene expression in PCA while the separation for BW groups was less evident, indicating that over time, the expression of genes converged across groups. Furthermore, the PERMANOVA analysis did not identify any distinct separation in gene expression profiles between BW groups at these later growth stages (*P* = 0.325 and *P* = 0.169, respectively, Figures 3B and 3C).

**Figure 4:**
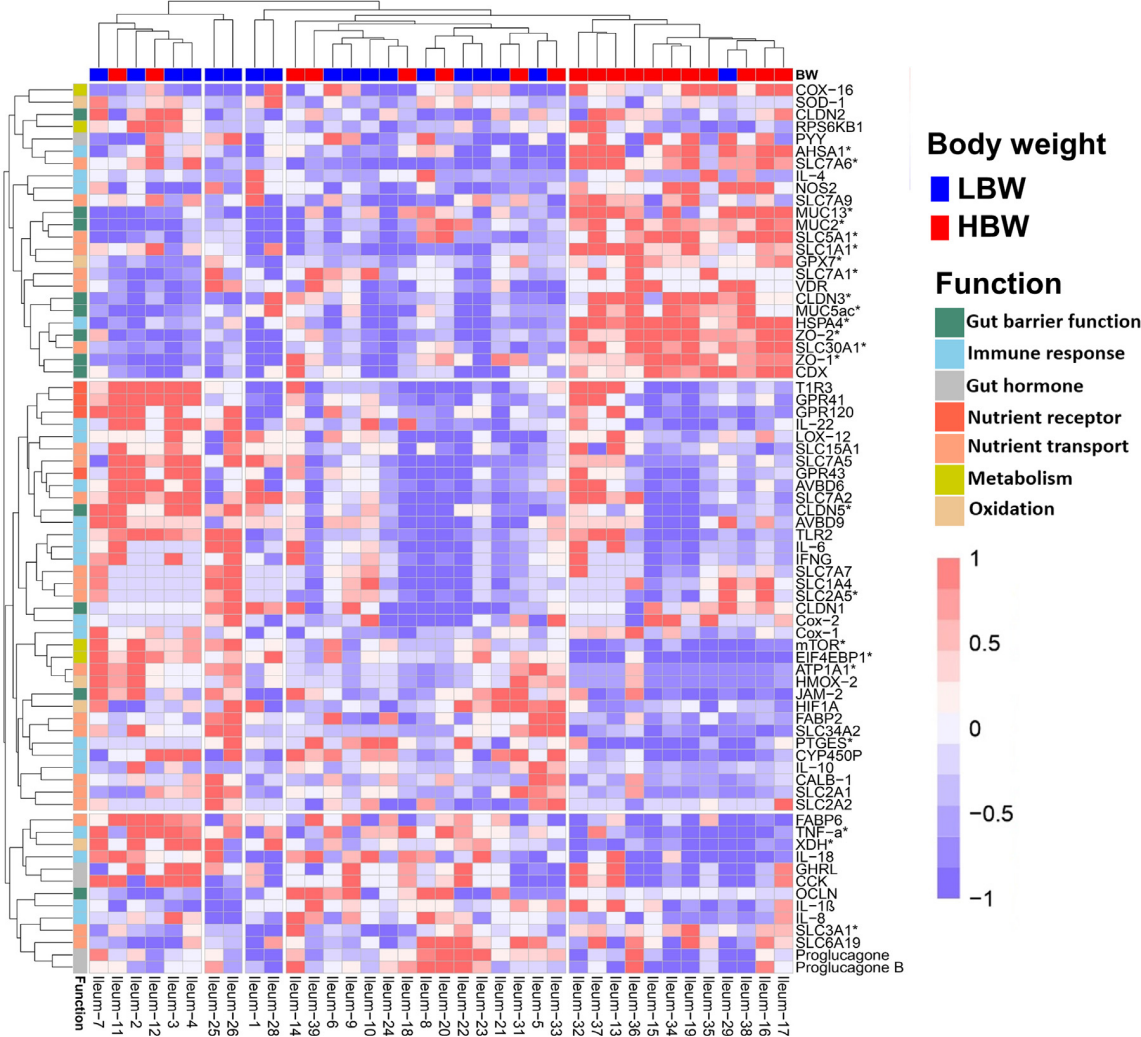
Two-way hierarchical cluster analysis showing the expression level of the genes analyzed in the ileum between low (LBW, n = 19) and high (HBW, n = 20) body weight groups on d 7. Samples are represented on the x-axis and genes on the y-axis. The red color indicates high expression while blue indicates low expression. Gene functions (y-axis) were labeled with different colors. The dendrogram on the left of the heatmap clusters genes with similar expression patterns, while the dendrogram on the top groups samples with similar gene expression profiles. Genes with (*) indicate significant differences between BW groups based on the univariate analysis (Student's *t*-test).

Two-way hierarchical cluster analysis (Figure 4, Supplementary file 1, Figure S1 and S2) revealed the overall variance in gene expression profiles among the samples from both BW groups on d 7, 14 and 38, respectively. On d 7, we identified three distinct clusters based on gene expression patterns, while five clusters were identified based on BW. The majority of the samples from the HBW group tended to cluster together and showed higher expression of genes within the first row cluster, which contains genes related to gut barrier function (*CLDN2, CLDN3, ZO-1, ZO-2, MUC2, MUC13, MUC5ac*), immune response (*AHSA1* and *HSPA4*), nutrient transport (*SLC1A1, SLC5A1, SLC7A1, SLC7A6, SLC7A9* and *SLC30A1*), gut hormone (***PYY***), metabolism (***COX-16***) and oxidation (***GPX7***). The HBW samples exhibited lower expression of genes in the other 2 row clusters. The two-way hierarchical clustering of samples and genes on d 14 and 38 was not as distinct, aligning with PCA and PERMANOVA findings. Neither the samples nor the genes showed clear clustering for BW groups or their biological functions, respectively.

### Differential Gene Expression Analysis

**Figure 5:**
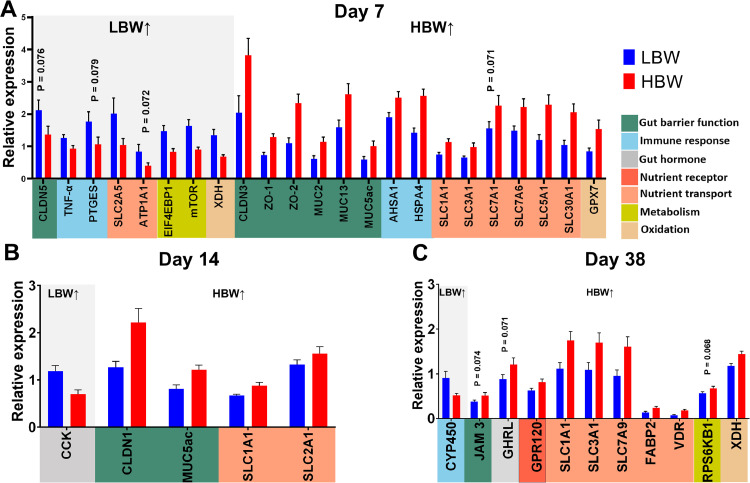
Significantly different genes between low (**LBW**) and high (**HBW**) groups on d 7 (A), 14 (B), and 38 (C). Genes that were significantly upregulated in the LBW group are shown on the left side of the bar plot under the gray color background, while genes that were significantly upregulated in the HBW group are shown on the left side (white background). Gene functions are labeled with various colors (x-axis). Statistical analysis was conducted using the Student's *t-*test with an FDR-adjusted *P* value < 0.05 to control for false discovery rate.

To further investigate the number of genes differentially expressed between BW groups, a univariate analysis approach using Student's t-test was performed. Genes with an FDR corrected *P*-value less than 0.05 were considered significantly different between groups and presented in Figure 5. On d 7, the HBW group showed higher expression of genes associated with gut barrier function, including *CLDN3* (*P* = 0.021), *ZO-1* (*P* = 0.009), *ZO-2* (*P* = 0.004), *MUC2* (P = 0.006), *MUC13* (P = 0.016), and *MUC5ac* (P = 0.026) (Figure 5A). In contrast, LBW group showed a tendency towards increased CLDN5 expression (*P* = 0.076). The LBW group exhibited higher expression of genes related to the immune response, such as *TNF-α* (*P* = 0.025) and a tendency for increased *PTGES* expression (*P* = 0.079). However, the HBW group showed increased expression of *AHSA1* and *HSPA4* genes (*P* = 0.015 and *P* = 0.001). Regarding nutrient transport, the HBW group demonstrated upregulation of various SLC genes, including *SLC1A1* (*P* = 0.004), *SLC3A1* (*P* = 0.025), *SLC7A6* (*P* = 0.031), *SLC5A1* (*P* = 0.005), and *SLC30A1* (*P* ≤ 0.001), while showing a decrease in *SLC2A5* (*P* = 0.041) and a tendency for decreased *ATP1A1* expresions (*P* = 0.072). The LBW group exhibited higher expression of the metabolism-related genes *EIF4EBP1* (*P* = 0.002) and *mTOR* (*P* = 0.002), as well as altered expression of oxidation-related genes, with increased *XDH* (*P* ≤ 0.001) and decreased *GPX7* (*P* = 0.025).

On d 14, the LBW group showed down-regulation of the gut barrier-related genes *CLDN1* (*P* = 0.010) and *MUC5ac* (*P* = 0.012), while showing higher expression of *CCK* (*P* ≤ 0.001), a gut hormone (Figure 5B). The HBW group exhibited upregulation of the nutrient transporter genes *SLC1A1* (*P* = 0.033) and *SLC2A1* (*P* = 0.048). On d 38, the HBW group tended to have increased expression of the barrier-related gene *JAM 3* (*P* = 0.074), the gut hormone *GHRL* (*P* = 0.071) and the nutrient receptor gene *GPR120* (*P* = 0.035) (Figure 5C). Additionally, the HBW chicks exhibited higher expression of nutrient transport genes, including *SLC1A1* (*P* = 0.041), *SLC3A1* (*P* = 0.031), *SLC7A9* (*P* = 0.017), *FABP2* (*P* = 0.017), and *VDR* (*P* = 0.009). In the oxidation and metabolism categories, *XDH* was significantly higher (*P* = 0.007), while *RPS6KB1* (*P* = 0.068) tended to be higher in HBW chickens. The LBW group demonstrated a higher expression of the immune-related gene *CYP450* (*P* = 0.021).

### Partial Least Square Regression Models

**Figure 6:**
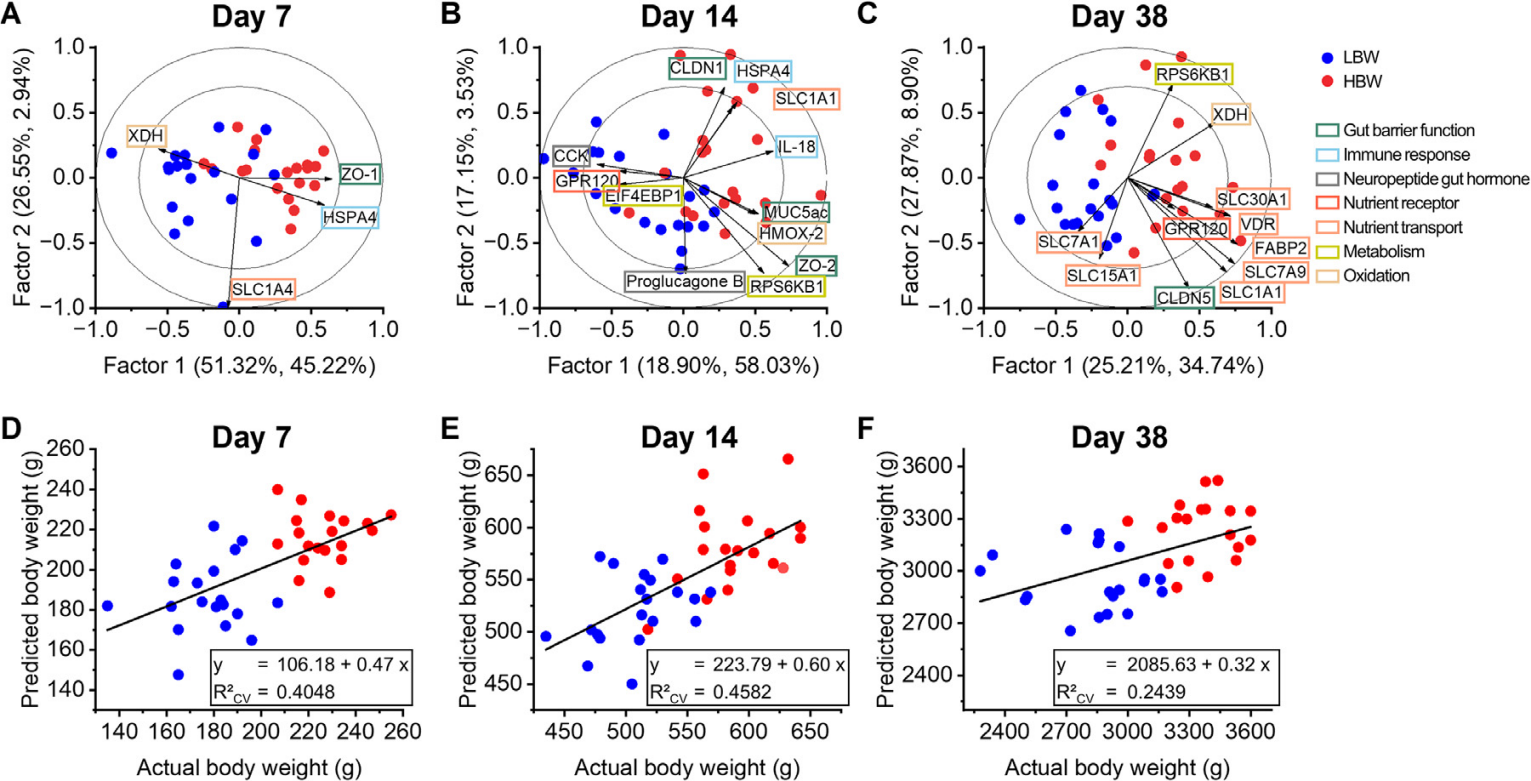
Partial least squares regression (**PLSR**) models illustrating the relationship between ileal gene expression and body weight in low (**LBW**) and high (**HBW**) body weight broilers on d 7 (A), 14 (B), and 38 (C). These models were obtained through a variable selection for variables with a variable of importance in projection (**VIP**) ≥ 1 for which the genes are labeled on the biplots. The colored frames around the genes denote gene function, while the outer and inner circles depict the 100 and 50% explained variance, respectively. The percentage of x and y variance per factor is presented in parentheses. Additionally, linear fit equations and R-squared values on the cross-validation sets (R^2^_CV_) for the PLSR models of d 7 (D), 14 (E), and 38 (F) are presented.

Following PCA, we acquired valuable insights into the overall gene expression patterns delineating between the BW groups. Furthermore, differential gene expression was determined for each day through a Student's *t*-test. This section employs targeted PLSR analysis to identify the most effective combination of key genes and their role in explaining the variance in BW between the two BW groups. The PLSR models identified the genes with VIP ≥ 1 as the most important discriminatory features between the groups on d 7, 14, and 38 (Figure 6). As a result, all three PLSR models were comprised of two factors based on the minimal root mean squared error of cross-validation (**RMSECV**). On d 7, the PLSR model identified a combination of four genes related to gut barrier function (*ZO-1*), immune response (*HSPA4*), nutrient transport (*SLC1A4*), and oxidation (*XDH*) as highly predictive of the BW phenotype (Figure 6A). The model yielded an R^2^_CV_ value of 0.4048, indicating that the expression of these 4 genes could explain 40.48% of the variance in BW (Figure 6D). On d 14, the PLSR model identified 12 genes as important predictors of BW, with an R^2^_CV_ of 0.4582, explaining 45.82% of the variability in BW (Figures 6B and 6E). The majority of the genes were upregulated in the HBW group and were related to gut barrier function (*CLDN1, ZO-2*, and *MUC5ac*), immune response (*HSPA4* and *IL-18*), nutrient transport (*SLC1A1*), metabolism (*RPS6KB1*), and oxidation (*HMOX-2*). However, gut hormone (*CCK* and *Proglucagon B*), nutrient receptor (*GPR120*), and metabolism (*EIFEBP1*) genes were identified as higher in the LBW group. By d 38, the PLSR model identified a combination of 11 genes as a discriminatory factor between BW groups, with an R^2^_CV_ of 0.2439 (Figure 6). A higher number of genes were identified in the HBW group compared to the LBW group, and the majority of these genes were related to the nutrient transport (*SLC1A1, SLC7A9, SLC30A1, FABP2* and *VDR*) as well as some genes from other categories such as gut barrier function (*CLDN5*), nutrient receptor (*GPR120*), metabolism (*RPS6KB1*) and oxidation (*XDH*). However, the predictive power of the gene expression data was lower at this late time point compared to the earlier days, but it still held the capacity to explain 24.39% of the variance in BW.

### Pearson's Correlation Between Body Weight and Growth Parameters, Visceral Organ Size, and Intestinal Structure and Function

**Figure 7:**
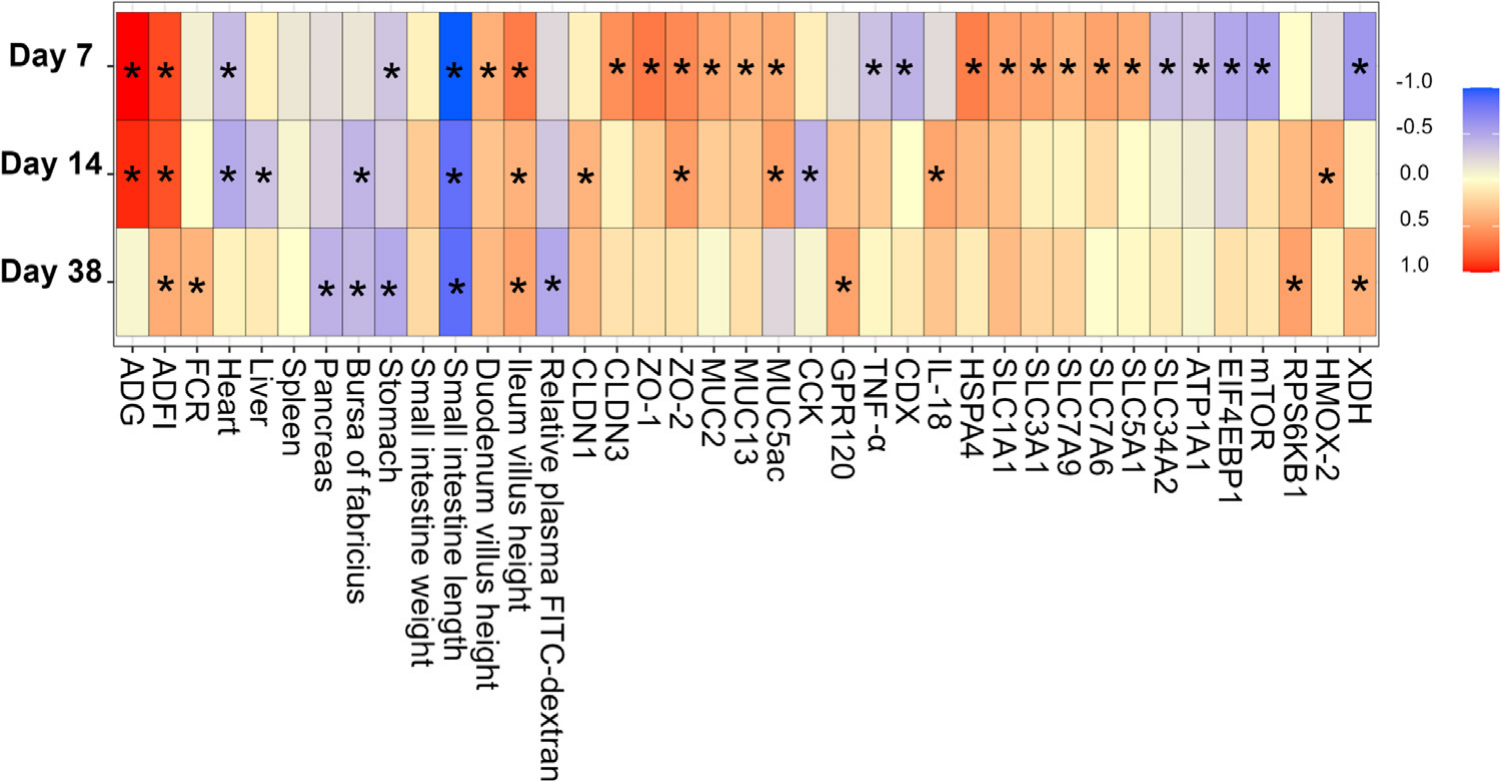
Pearson's correlation between body weight (**BW**) and growth parameters, visceral organs, and ileum gene expression levels on d 7, 14, and 38. Only significant correlations with *P* < 0.05 and |R| > 0.30 are showed and indicated with an asterisk (*).

Figure 7 shows Pearson correlations between BW and growth parameters, visceral organ size, and intestinal structure and function across 3 time points, with the highest number of significant correlations found on d 7. Positive correlations were observed between BW, ADG, and ADFI on d 7 and 14, while FCR exhibited a significant positive correlation with BW only on d 38. BW showed a positive correlation with ileal VH but was negatively correlated with relative small intestine length throughout the study period. Relative heart weights were negatively correlated with BW during the first 2 wk, and the relative weights of the bursa of Fabricius demonstrated a negative correlation with BW on d 14 and 38. On d 14, the liver's relative weight was negatively correlated with BW, while the stomach's relative weight showed negative correlations on d 7 and 14. Additionally, the pancreas’ relative weight demonstrated a negative correlation with BW on d 38. Similarly, the correlation between BW and relative plasma FITC-dextran was negative on d 38. BW on d 7 correlated positively with the expression of genes related to gut barrier function (*CLDN3, ZO-1, ZO-2, MUC2, MUC13*, and *MUC5ac*), and nutrient transporters (*SLC1A1, SLC3A1, SLC7A9, SLC7A6*, and *SLC5A1*). In contrast, negative correlations were observed with genes associated with the immune response (*TNF-α*), metabolism (*EIF4EBP1* and *mTOR*), and oxidation (*XDH*). Certain genes associated with nutrient transporters, such as SLC34A2 and ATP1A1, showed negative correlations with BW. On d 14, BW correlated positively with genes related to barrier function (*ZO-2* and *MUC5ac*), immune response (*IL-18*), oxidation (*HMOX-2*), and negatively with the expression of the digestive hormone-related gene *CCK*. On d 38, BW correlated positively with gene expression related to nutrient receptors (*GPR120*), metabolism (*RPS6KB1*), and oxidation (*XDH*).

## DISCUSSION

The categorization of broilers based on their d 7 BW relative to the flock mean revealed several important biological concepts. Despite being subjected to identical management practices, broilers with lower initial BW failed to exhibit any catch-up growth, suggesting that d 7 BW is a strong indicator of chicks' growth potential and slaughter weight (Wilson, 1991). The HBW group demonstrated higher ADG compared to the LBW group during the starter and grower periods, while their FCR remained comparable. This suggests that the discrepancy in ADG is primarily due to higher voluntary feed intake by the HBW group rather than more efficient utilization of dietary nutrients, suggesting differential feed intake behavior as a discerning factor in elucidating the growth patterns that contributed to divergent weight gain. In agreement, a similar study in pigs linked the growth lag in LBW piglets to reduced feed intake, which decreased the nutrient supply for pre- and postweaning growth (De Vos et al., 2014).

Heavier relative visceral organs require higher nutrients, leading to increased maintenance energy expenditures and lower chicken performance (Kolakshyapati et al., 2020). Consistent with these findings, the LBW group had significantly heavier hearts during the early growth stages and larger bursa of Fabricius in the later stage. Longer small intestines are commonly believed to enhance nutrient absorption and facilitate growth in chickens. Contrary to this assumption, our findings observed a shorter small intestine length in HBW chickens, suggesting the presence of a metabolic energy-saving mechanism in these chickens, wherein nutrients may be partitioned more efficiently towards growth and weight gain, rather than diverting resources to maintain a longer small intestine (Canas et al., 1982; Cant et al., 1996). Despite the shorter intestinal length, the increased development of villi in HBW chickens compensated for this reduction. The HBW group demonstrated higher VH and VH:CD ratios, which could provide an expanded surface area underlying a higher nutrient requirement, thereby contributing to their accelerated growth rate (Abdel-Kafy et al., 2022). Increased weights of the stomach, liver, and pancreas are indicators of improved digestive efficiency (Ravindran and Reza Abdollahi, 2021; Hikawczuk et al., 2023). Pearson correlation analyses identified these digestive organs as being heavier in the LBW group at various growth stages, potentially representing a compensatory mechanism to enhance digestive capacity and address growth challenges within their GIT.

The GIT, constantly exposed to a variety of foreign antigens, necessitates rapid mucosal restoration mechanisms in the event of tissue damage (Chen et al., 2015). Increased inflammation can destroy the intestinal structure and impair TJ integrity (Chen et al., 2015; Gilani et al., 2016), facilitating the translocation of antigens and toxins into the systemic circulation. In our study, in vivo gut permeability and gene expression results indicated that there was a disrupted gut barrier function in the LBW chickens, with consistently and significantly decreased relative mRNA expression of TJ genes, including *CLDN1, CLDN3, CLDN5, ZO-1*, and *ZO-2* compared to the HBW chickens. Several studies have linked compromised intestinal TJs in chickens to impaired health and performance (Murai et al., 2018). In contrast, the HBW chickens demonstrated higher expression of mucin producing genes, including *MUC2, MUC13*, and *MUC5ac*, likely reflective of the strong ability for more efficient clearance of bacteria and a robust protective barrier against pathogen colonization (Forder et al., 2012; Santos et al., 2024). This is corroborated by the fact that the *MUC2* gene expression has been used as a marker for gut health in poultry and other species (Forder et al., 2012), and has been shown to reduce *Salmonella* adhesion in the jejunum (Santos et al., 2024).

The HBW chickens exhibited increased relative mRNA expression of *SLC2A1* and *SLC5A1*, which are linked to glucose efflux. Previous studies have linked higher mRNA levels of glucose transporters with an increased BW in chickens (Zhang et al., 2013; Abdel-Kafy et al., 2022). The LBW chickens demonstrated increased expression of the fructose transporter *SLC2A5* on d 7, suggesting an adaptive response to address potential nutritional challenges due to lower feed intake. Additionally, the HBW chickens consistently demonstrated higher mRNA levels of various amino acid transporters across different growth stages, including *SLC1A1, SLC3A1, SLC7A1, SLC7A6*, and *SLC7A9*, indicating their capability to support rapid growth and weight gain through increased amino acid uptake. The *FABP2* gene, known for its role in lipogenesis and fatty acid transport in broilers (Hu et al., 2010), is also recognized as a marker for gut barrier health and epithelial content in humans and pigs (Chen et al., 2015). In the present study, gene expression of *FABP2* was higher in HBW chickens on d 38, suggesting enhanced free fatty acid absorption, increased epithelial cell content, and a strengthened intestinal barrier function. Furthermore, upregulation of genes like the vitamin D receptor (*VDR*) and *SLC30A1* in HBW chickens suggests enhanced absorption of crucial nutrients like calcium and zinc, important for intestinal function and overall growth.

Previous studies have reported higher relative gene expression of pro-inflammatory mediators, including *TNF-α* in LBW chickens compared to their HBW counterparts (Zhang et al., 2022), with overwhelming production of pro-inflammatory cytokines being indicative of an inflammatory state (Yang et al., 2015). In line with these findings, our study found significantly higher gene expression of proinflammatory cytokine *TNF-α* and a tendency for increased *PTGES* on d 7 in LBW chicken's ileum. *TNF-α* is a pleiotropic cytokine, and has been shown to potentially affect barrier function by downregulating tight junction proteins (Wang et al., 2005). *PTGES* is a fundamental gene required for the synthesis of prostaglandins, which are well-known inflammatory mediators (Niu et al., 2019). As such, the elevation of *PTGES* has been linked to intestinal inflammation (Keita et al., 2010). In addition to cyclooxygenase pathway, cytochrome P450 (CYP450) enzyme provide an alternative pathway for the metabolism of arachidonic acid, a polyunsaturated fatty acid, into eicoanoides (Wang et al., 2021). These eicosanoids, including prostaglandins, thromboxanes, and leukotrienes, modulate immune cell activity and are generally considered pro-inflammatory molecules due to their potent effects on inflammation, oxidative stress, and immune response stimulation (Mendoza et al., 2021; Zhang et al., 2023). On d 38, the LBW chickens exhibited higher expression of *CYP450* gene compared to their heavier counterparts, suggesting potential implications for eicosanoid production and inflammatory processes. This finding aligns with previous research linking growth restriction of chickens to a predisposition for pro-inflammatory states and an increased risk of inflammatory disorders (Zhang et al., 2022). The increased immune response in LBW chickens may not solely result from active infection but rather suggests a basal immune system activation, possibly adapting to counteract an imbalanced gut microbiota, rich in opportunistic pathogenic bacteria. Indeed, the gut microbiota plays a pivotal role in shaping the host's immune response (Kogut et al., 2020), with previous studies reported an imbalanced microbiome composition in LBW chickens, characterized by a higher abundance of opportunistic pathogens like *Escherichia-Shigella* (Akram et al., 2024). Gram-negative pathogenic bacteria contribute to the release of lipopolysaccharides that induce the expression of inflammatory mediators (Lin et al., 2020). This increased immune response can place a significant nutritional burden on the host (Willson, 2017), diverting nutrient resources toward immunity at the cost of rapid growth. On the other hand, *AHSA1* gene, which encodes a protein responsible for activating the ATPase activity of the heat shock protein (**HSP**) 90 chaperone, was upregulated in the HBW group on d 7. This protein plays a crucial role in the stress response and regulation of Hsp90-dependent cellular pathways in broiler chickens (Criado-Mesas et al., 2021). HBW group also exhibited a higher expression of the *HSPA4* gene, a member of the HSPs, on d 7. HSPs play a critical role in gut health and immune regulation, acting as molecular chaperones for maintaining gut epithelium integrity and effective intestinal barrier function (van Eden, 2014). The univariate analysis revealed a higher expression of *GPR120* mRNA on d 38 in the HBW group, a receptor that binds unsaturated long-chain fatty acids and derivatives (Ulven and Christiansen, 2015). *GPR120* monitors fatty acid concentrations in gastrointestinal and oral tissues (Kawabata et al., 2022), and is also highly expressed in adipose tissues and pro-inflammatory macrophages. Its activation mediates anti-inflammatory effects, which reduces the inflammatory signaling responses induced by lipopolysaccharide and *TNF-α* cytokine (Oh et al., 2010). Thus, the upregulation of this gene in HBW chickens further confirms their enhanced capacity to regulate lipid metabolism and inflammatory processes and maintain gastrointestinal homeostasis.

Feed intake is known to be strongly correlated with weight gain in broilers (Skinner-Noble and Teeter, 2004). Differential expression of gut hormones, such as the increased proglucagon B and *CCK* in the LBW group on d 14 and the tendency for higher *GHRL* in the HBW group on d 38, may have contributed to the divergent feed intake behaviors and subsequent growth variation between BW groups. *Proglucagon B*, which was identified as a predictive gene for LBW by PLSR analysis on d 14, is a precursor for glucagon, glucagon-like-peptide-1 (**GLP-1**), and glucagon-like-peptide-2 (**GLP-2**), known to have pronounced effects on appetite and food intake regulation (Lafferty et al., 2021). Glucagon reduces BW and adiposity in humans by suppressing appetite and modulating lipid metabolism (Scott and Bloom, 2018). CCK hormone serves as a satiation signal and contributes to the feeling of fullness and satisfaction after eating (Miller, 2019), might be leading to a reduction in feed intake in LBW group. Studies have shown that administering CCK reduces feed intake in chickens (Tachibana et al., 2012), while inhibiting CCK-A receptors promotes growth and increases BW (Dunn et al., 2013). The HBW group had higher feed intake than LBW group, which is corroborated by previous findings (Fang et al., 2007; Honda et al., 2017) reporting higher GHRL, known as the "hunger hormone," is involved in increasing feed intake and weight gain in chickens by transmitting the hunger signal to the brain before feed intake. *mTOR* and *EIF4EBP1* are central regulators of cellular processes such as protein synthesis, cell growth, and metabolism (Criado-Mesas et al., 2021). Their increased expression in the LBW group on d 7 suggests an attempt to stimulate cellular growth and proliferation as a response to developmental challenges in early life.

Modern fast-growing broilers are highly susceptible to the detrimental effects of excessive reactive oxygen species (**ROS**) resulting from cellular metabolism, which contributes to intestinal oxidative stress (Pesti-Asbóth et al., 2023). These ROS adversely affect the antioxidant system in the gut and lead to health problems (Mishra and Jha, 2019). The HBW chickens exhibited higher gene expression of antioxidant enzymes, including *GPX7* on d 7, *HMOX-2* on d 14, and *XDH* on d 38, indicating their ability to combat excess free radicals and maintain homeostasis through an activated antioxidation system. Interestingly, *XDH* exhibited dual behavior, with higher expression in the LBW chickens on d 7 but higher in the HBW chickens on d 38, suggesting that some genes may play different roles across growth stages.

The univariate approach with the Student's t-test provided a list of individual genes exhibiting significant expression changes but did not account for the potential combined effects of multiple genes. The PLSR model's strength lies in its ability to identify combination of co-expressed genes whose expression highly correlates with the underlying observed phenotypes, regardless of the expression level. On d 7, the PLSR model selected only four genes (*ZO-1, HSPA4, SLC1A4* and *XDH*) as the most important discriminatory features, while adding extra genes did not improve model performance. This suggests that these 4 genes represented a concise set of biomarkers effectively capturing the underlying molecular differences between BW groups in the first week. In contrast to d 7, the PLSR models for d 14 and 38 required a larger number of genes, indicating more information from different genes was needed to explain the BW variance at later growth stages. The PLSR analysis revealed an early genetic signature explained by genes involved in gut barrier function (*ZO-1*), immune response (*HSPA4*), and oxidation (*XDH*) that transitioned towards a lasting profile of genes regulating nutrient transport (*SLC1A1, SLC7A1, SLC15A1, SLC7A9, SLC30A1, FABP2* and *VDR*), nutrient receptor (*GPR120*), gut hormone (*CCK* and *Proglucagone B*) and metabolism (*RPS6KB1* and *EIF4EBP1*) as determinants of BW phenotypes over time. Interestingly, genes associated with gut barrier integrity and oxidation remained as consistent predictors of BW phenotype across all time points. The PLSR models based on gene expression data explained 40% and 45% of the BW variance on d 7 and 14 by identifying 4 and 12 key genes, respectively. However, by d 38, the model's predictive accuracy decreased substantially, possibly due to the convergence of growth rate differences between BW groups during the late growth stage. Nevertheless, the robust predictive accuracy of the PLSR models on d 7 and 14 offers a valuable tool for early identification of the growth potential, informing strategies to improve broiler flock uniformity.

The study aimed to standardize conditions from placement to slaughter for all broiler birds, yet significant variations in growth rates were observed. Egg age and storage conditions were similar for all the chicks in our study as they originated from one flock, of 40-wk old parents, and all eggs were placed in 1 incubator, hence minimizing factors related to parent flock and incubation. Hatch weight is a good predictor of subsequent performance, with heavier chicks typically showing improved growth rates (Chen et al., 2013). On d 1, chicks had similar BW with low variance, indicating minimal differences in hatch weights. Despite the low genetic variations within highly inbred broiler lines, residual heterozygosity and genetic polymorphisms yet exists, which may contribute to phenotypic variation among chicks within a shared environment (Reyer et al., 2015). Additionally, variability in gut microbiota composition among individuals within a flock can influence nutrient digestion, absorption, gut integrity, and immune function, thereby impacting growth trajectories (Akram et al., 2024). Moreover, epigenetic changes induced by early-life conditions such as incubation conditions, chick transportation and environmental stress can impact bird performance by altering physiological mechanisms and metabolic pathways. LBW and HBW chicks were housed separately with *ad-libitum* feed and ample feeder space to reduce competition. However, observed differences in feed intake behaviors among BW categories, influenced by unknown physiological and environmental factors, may have altered nutrient availability and signaling molecules in the gut, subsequently impacting gut health and host responses.

Given the significant impact of early-life differences in gut function and gene expression, implementing targeted strategies during this critical period is essential for improving GIT development, gut health, and feed intake behavior, especially in LBW chickens. Such strategies can be implemented through management practices like providing early access to feed and making nutritional and dietary modifications. These approaches may help address gut health deficiencies in LBW chickens and thereby reduce BW heterogeneity.

## CONCLUSIONS

The results demonstrated that differences in feed intake and gut-related characteristics contributed to the observed variation in BW of the chickens reared under uniform management conditions. HBW birds exhibited more efficient digestive physiology characterized by shorter relative intestinal length but higher absorptive capacity (longer VH, greater VH:CD ratio), and enhanced expression of genes involved in maintaining gut barrier integrity and nutrient transport. In contrast, the LBW group demonstrated more energy-intensive visceral organ development, activation of pro-inflammatory response genes, and increased intestinal permeability across various growth stages, potentially leading to higher maintenance energy requirements. The findings further suggest divergent hormonal regulation of appetite and feed intake as a significant driver of the observed variation in growth rates among broilers. PLSR predictive models identified combinations of genes as highly predictive of BW phenotypes, with high model predictive power during early growth stages. These findings suggest that the divergence in BW outcomes is driven, at least in part, by differences in the gene expression of various intestinal functions between birds, offering insights into the molecular mechanisms governing growth. The insights gained in this study shed light on the underlying gut-related regulatory mechanisms involved in broiler growth rates and also highlighted the importance of tailored management practices to optimize production efficiency and flock uniformity.

## DISCLOSURES

The authors declare no conflicts of interest.

## Appendix Supplementary materials

Image, application 1

## Footnotes

Supplementary material associated with this article can be found in the online version at doi:10.1016/j.psj.2024.104158.
