# Supplementary material for: Linking gastrointestinal tract structure, function, and gene expression signatures to growth variability in broilers: a novel interpretation for flock uniformity
Source: Poult Sci. 2024 Aug 2;103(10):104158. doi: 10.1016/j.psj.2024.104158 (PMC11387703; doi:10.1016/j.psj.2024.104158)
Supplement: Supplementary file 1 [file mmc1.pdf]

Linking gastrointestinal tract structure, function, and gene expression signatures to growth variability in broilers a novel interpretation for flock uniformity

Muhammad Zeeshan Akram[1,2], Ester Arévalo Sureda[1], Matthias Corion[1], Luke Comer[1], Nadia Everaert[1, Ŧ]

[1]Nutrition and Animal-Microbiota Ecosystems Laboratory, Department of Biosystems, KU Leuven, 3000-Heverlee, Belgium

[2]Precision Livestock and Nutrition Unit, Gembloux Agro-Bio Tech, University of Liège, Gembloux, Belgium,

[Ŧ]Corresponding author: Nadia Everaert

Table S1: Composition (%) of the feed offered to broilers during starter, grower, and finisher phases.

Ingredients %	Starter<br>1 – 14 days	Grower<br>15 – 28 days	Finisher<br>29 – 38 days
Maize	15.0	10.0	5.0
Wheat (fine)	33.73	39.20	48.88
Wheat (coarse)	5.0	10.0	10.0
Maize gluten	2.88	3.32	0
Soy oil	4.61	5.02	5.32
Soybean meal	29.86	24.79	25.42
Sunflower meal	2.5	2.5	0
Oat hull (coarse)	1.0	0	0
Sodium bicarbonate	0.23	0.23	0.25
Salt	0.16	0.16	0.16
Choline 75%	0.09	0.09	0.09
[1]Premix	0.30	0.30	0.30
Limestone	1.44	1.31	1.19
Monocalcium Phosphate	1.13	0.92	0.70
Lysine	0.55	0.52	0.30
Methionine	0.26	0.22	0.22
L-Threonine	0.14	0.12	0.09
L-Valine	0.02	0	0
[2]Avi-Deccox	0.05	0.05	0
L-Arginine	0.03	0.02	0.02
Palm oil spray	1.0	1.22	2.03
[3]Phytase	0.01	0.01	0.01
Chemical Composition			
Metabolizable energy (kcal/kg)	3000	3100	3200
Digestible lysine (%)	1.28	1.15	1.02
Crude protein (%)	23.0	21.5	19.5
Calcium (%)	0.96	0.87	0.78
Available phosphorus (%)	0.48	0.44	0.39
Sodium (%)	0.14	0.14	0.14
Chloride (%)	0.18	0.18	0.16
Potassium (%)	1.0	0.90	0.90

[1]Provided per kg feed: Vit A 10.0 IU, Vit D3 2750 IU, 25-hydroxycholecalciferol 0.06 mg, Vit E 90 mg, Copper 15 mg, Iron 15 mg, Manganese 85 mg, Zinc 50 mg, Iodine 2 mg, Selenium 0.4 mg.

[2]Provided per kg feed: 30.3 mg decoquinate.

[3]Provided per kg feed: 500 FTU.

Table S2: Primers used for high-throughput qPCR, and a brief description of their main function. There are 79 target genes and 13 reference genes in total.

Function	Gene	Full name	Description	Primers (5' → 3')	Accession no	Reference
**Barrier function**	*CLDN1*	Claudin-1	Transmembrane protein of tight junction (TJ)	F: CTTCATCATTGCAGGTCTGTCAG<br>R: AAATCTGGTGTTAACGGGTGTG	NM_0010136 11.2	(Zanu et al., 2020)
	CLDN2	Claudin-2	Transmembrane protein of TJ	F: ACTGCAGCTGCCCTCGGT<br>R: AAGCTTCACCCTGCTGCTGT	NM_0012776 22.1	This study
	CLDN3	Claudin-3	Transmembrane protein of TJ	F: GCCAAGATCACCATCGTCTC<br>R: CACCAGCGGGTTGTAGAAAT	NM_204202. 2	(Barekatain et al., 2019)
	CLDN4	Claudin-4	Transmembrane protein of TJ	F: CTGTGCCGGGACACTGAATG<br>R: TCCTCCACAGTGGTGTTTGG	XM_0036423 82.6	This study
	CLDN5	Claudin-5	Transmembrane protein of TJ	F: GTCCCAGAAGCGGGAGATAG<br>R: CGAGTACTTGACGGGGAAGG	NM_204201. 2	This study
	OCLN	Occludin	Protein of TJ involved in both inter-membrane and paracellular diffusion of small molecules	F: ACGGCAGCACCTACCTCAA<br>R: GGGCGAAGAAGCAGATGAG	NM_205128. 1	(Zanu et al., 2020)
	ZO-1	Zonula Occludens-1	Scaffold proteins that form part of the cytoplasmic plaque of TJ	F: ACCACAAGGAGCCATTCCAG<br>R: GTGAGGCCCACACATTACCA	XM_0406806 24.2	This study
	ZO-2	Zonula Occludens-2	Scaffold proteins that form part of the cytoplasmic plaque of TJ	F: GCCCAGCAGATGGATTACTT<br>R: TGGCCACTTTTCCACTTTTC	XM_0406554 19.2	(Barekatain et al., 2019)
	JAM-2	Junctional Adhesion Molecule-2	Transmembrane protein of TJ	F: GGTACTTGGGGGTCTTCTGC<br>R: TGTGCTTGCAACTAAGAATAGCC	NM_0013971 41.1	This study
	JAM-3	Junctional Adhesion Molecule-3	Transmembrane protein of TJ	F: CCAGAGTGTTGAGCTGTCCT<br>R: AGAATTTCTGCCCGAGTTGC	XM_417876	(Proszkowiec-Weglarz et al., 2020)
	MUC2	Mucin-2	Secretory mucine important in the establishment of the mucus layer	F: CCCTGGAAGTAGAGGTGACTG<br>R: TGACAAGCCATTGAAGGACA	XM_0469422 97.1	(Zanu et al., 2020)
	MUC13	Mucin-13	Transmembrane mucine that plays a role in cell signalling pathways	F: CCAGGCACCAGAAGTGCTAA<br>R: TGCGTACTGATGCACGTAGT	XM_0036415 85.6	This study
	MUC5ac	Mucin-5AC	Mucin 5ac	F: TGTGGTTGCTATGAGAATGGA<br>R: TTGCCATGGTTTGTGCAT	XM_0036413 22.2	(Forder et al., 2012)
	CDX	Caudal Type Homeobox Transcription Factor	Intestinal tract development	F: ACAGCTGTCCCCTAATGCAC<br>R: TCCTTTGTCCTCGTCTTGCC	NM_204676. 3	This study
**Gut hormone**	*GHRL*	Ghrelin	Ghrelin: Hunger hormone. Induces motor activity in the intestinal tract	F: AACTGCTCTGGCTGGCTCT<br>R: CTCCCTCTGTTTCATCTGTAT	XM_0469261 85.1	(He et al., 2018)
	Proglucagone	Proglucagon	Precurose of GLP-1	F: CACAAGGCACATTCACCAGT<br>R: TTCTTTGGCAGCTTGACCTT	NC_052538.1	(Herwig et al., 2020)
	Proglucagone B	Proglucagon related protein	Precursor of GLP-1	F: CACAAGGCACATTCACCAGT<br>R: TGGTATTCTCCCAAAAGGTCTC	NC_052538.1	(Herwig et al., 2020)
	PYY	Peptide YY	Peptide tyrosine tyrosine. feed intake regulatory hormone	F: AGGAGATCGCGCAGTACTTCT<br>R: TGCTGCGCTTCCCATACC	NM_0013611 82.2	(Herwig et al., 2020)
	CCK	Cholecystokinim	Feed intake regulatory hormone	F: GAAGGTAGGGAGCGGCAC<br>R: TCGGAAAAGGGGGAAAACGA	XM_0152813 32.4	(Song et al., 2012)
**Nutrient receptors**	*T1R1*	Taste Receptor Type 1 Member 1	Taste receptor type 1 member 1	F: GTGTCATCCCCACAACCAA<br>R: CACCACTGCCTCAAAGAAGG	XM_425734. 4	(He et al., 2018)
	T1R3	Taste Receptor Type 1 Member 3	Taste receptor type 1 member 3	F: CATTACCGTCTTCGCCACTC<br>R: CTCTGTTCAAATCGGGCTTC	XM_425740. 3	(He et al., 2018)
	GRP43	G-protein coupled receptor 43	FFAR2 – activated by short chain fatty acids	F: AGGGAATCCGGGATGGAGAA<br>R: ACGCAGTCAGGTTGGTTCAA	NM_0013184 30.1	This study
	GPR41	G-protein coupled receptor 41	FFAR3 – activated by short chain fatty acids	F: GAAGGTGGTTTGGGAGTGAA<br>R: CAGAGGATTTGAGGCTGGAG	XM_427629	(Zhang et al., 2021)
	GPR120	G-protein coupled receptor 120	FFAR4 – activated by medium-chain and unsaturated long-chain FFAs	F: ACTTCACTGCTTTGCCTCAGT<br>R: CCAGTACAAGTGGAGGGTTCA	XM_0406754 55.2	This study

	IL-4	Interleukin-4	Cytokine that induces differentiation of naive helper T cells (Th0 cells) to Th2 cells	F: TTATGCAAAGCCTCCACAATTG<br>R: GTGGGACATGGTGCCTTGAG	XM_046900385.1	(Criado-Mesas et al., 2021)
	IL-6	Interleukin-6	Humoral immunity related genes	F: CTCGTCCGGAACAACCTCAA<br>R: GGAGAGCTTCGTCAGGCATT	NM_204628.2	This study
	IL-8	Interleukin-8	Secreted in response to pathogenic bacteria infection or specific inflammatory cytokines	F: AGATGTGAAGCTGACGCCAA<br>R: GAGCTGAGCCTTGGCCATAA	NM_205498.2	This study
	IL-10	Interleukin-10	Anti-inflammatory cytokine produced by activated macrophages and T cell	F: CTGAGGGTGAAGTTTGAGGAAAT<br>R: AGCCAAAGGTCCCCTTAAACTC	NM_001004414.4	(Criado-Mesas et al., 2021)
	IL-18	Interleukin-18	Pro-inflammatory cytokine. primarily produced by macrophages. targeting T helper type-1 (Th1) cells	F: CTCCTCCACACAGCAACACA<br>R: ATGCAGTTGGCCACTTCTGT	XM_046932259.1	This study
	IL-22	Interleukin-22	Commonly used as marker of inflammation involved in T-lymphocytes activation	F: ACATCAGGGAGAACAACCGC<br>R: TGCCACATCCTCAGCATACG	NM_001199614.1	This study
	IL-1β	Interleukin-1β	Mediator of the inflammatory response and involved in cellular processes	F: CGCTACACCCGCTCACAGT<br>R: GCAATGTTGAGCCTCACTTTCTG	XM_046931582.1	(Criado-Mesas et al., 2021)
	TLR2	Toll-like receptor 2	Transmembrane receptor for the recognition of gram positive bacteria	F: AGGCGATCCCAAGAGGTTC<br>R: TTTCCCAAAACATCTGCTGTTG	XM_046915412.1	(Criado-Mesas et al., 2021)
	TLR4	Toll-like receptor 4	Transmembrane receptor for the recognition of gram negative bacteria	F: CAGTCCGTGCCTGGAGGT<br>R: TTGAGCTTAGCAATTTCAGACTGTTG	NM_001030693.2	(Criado-Mesas et al., 2021)
**Immune response**	*TNF-α*	Tumor Necrosis Factor alpha	Regulation of the host immunity against multiple pathogens	F: TTGCGAGGGGAGAGGAGAAA<br>R: GTCAGTACCGCGTCGTCTTT	XM_046927261.1	This study
	AHSA1	Activator of Hsp90 ATPase	Co-chaperone activator of HSP90	F: GGGGAAGCCTCCATCAACAA<br>R: TCACTCCTGTGGTCGAGGT	XM_040672816.2	This study
	AvBD6	Avian β-defensin 6	Avian defense involved in antimicrobial functions and protecting the gut epithelium	F: CTTGCTGTGTGAGGAACAGGTG<br>R: TTTGGTAGTTGCAGGCAGGAT	NM_001001193.1	(Criado-Mesas et al., 2021)
	AvBD9	Avian β-defensin 9	Avian defense with antimicrobial properties and other cellular functions	F: CTGAGACCTCACTGACCACG<br>R: GTGCTCCCAGGACTCTTCAC	NM_001001611.3	(Criado-Mesas et al., 2021)
	HSPA4	Heat Shock Protein Family A Member 4	Member of HSP proteins and play a prominent role in repair and protection of the intestinal environment	F:TGAGACTAATAAATGAATCAACTGCAGT<br>R: CCCCATATCCACAAAAACAACA	XM_046927108.1	(Criado-Mesas et al., 2021)
	IFNG	Interferon gamma	Host defense for combating against the intracellular pathogens including Salmonella	F: ACCTTCCTGATGGCGTGAAG<br>R: CTGAAGAGTTCATTCGCGGC	NM_205149.2	This study
	NOS2	Nitric Oxide Synthase 2	Induce the development of Th1 type of IR in infections	F: CTCCAGCAGAGCTTCTACC TCAA<br>R: GCCAGGTGCTCTTCTATTT TTAATTC	NM_204961.2	(Criado-Mesas et al., 2021)
	PTGES	Prostaglandin E synthase	Intestinal inflammatory factor	F: GGCTCTGAGGACAATGCAGA<br>R: CCAGAGGAGAGCACAGCAAA	XM_046928949.1	This study
	Cox-1	Cyclooxygenase-1	Cyclo-oxygenase 1	F: GCGCATCAGTAGACCTAGCC<br>R: TGGTATTGTGACAGTGCGGG	JX160009.1	(Konieczka et al., 2019)
	Cox-2	Cyclooxygenase-2	Cyclo-oxygenase 2	F:ATTCCTGACCCACAAGGCAC<br>R: AGTCAACCCCATGGCCGTAA	NM_001167719	(Hollemans et al., 2020)
	Lox-12	Lipoxygenase-12	Lipo-oxygenase	F: CTGATTACGCCGTGCTGGAT<br>R: ATTGGGGCACACAGGAATGT	XM_015274997.1	(Konieczka et al., 2019)
	CYP450	Cytochrome P450	Cytochromes P450	F: ACCACTTCTGGAAGGAGGGA<br>R: CGCTCTCGTAGACACCCAAC	D49803.1	(Konieczka et al., 2019)
	SLC15A1	Solute Carrier Family 15 Member 1	Peptide transporter-1	F: CAGGATTTCCCTGTGTCAGGT<br>R: GCAGCGTGGACAAGTATGG	XM_046906441.1	This study
**Nutrient transport**	*SLC1A1*	Solute Carrier Family 1 Member 1	Excitatory amino acid transporter	F: TGCTGCTTTGGATTCCAGTGT<br>R:AGCAATGACTGTAGTGCAGAAGTAATATATG	XM_046936555.1	(Su et al., 2014)
	SLC1A4	Solute Carrier Family 1 Member 4	Neutral amino acid transporter by ASC system	F: ACAGCAAGCTGTGGTCAGAA<br>R: TCTCCCAGAATGCAATCACAGT	XM_046914471.1	This study
	SLC3A1	Solute Carrier Family 3 Member 1	Protein related to neutral amino acid transporter	F: CCCGCCGTTCAACAAGAG<br>R: AATTAAATCCATCGACTCCTTTGC	XM_040667709.2	(Su et al., 2014)

	SLC7A9	Solute Carrier Family 7 Member 9	Na+-independent neutral/cysteine. cationic amino acid exchanger	F:CAGTAGTGAATTCTCTGAGTGTGAAGCT<br>R: GCAATGATTGCCACAACTACCA	XM_046925529.1	(Su et al., 2014)
	SLC6A19	Solute Carrier Family 6 Member 19	Na+-dependent neutral amino acid transporter	F: CCAGAGGGCAATGTAACCCA<br>R: AAGGCTAAGCCGGTTCCTTC	XM_040663289.2	This study
	SLC7A1	Solute Carrier Family 7 Member 1	Transport lysine. arginine. and histidine	F: CAAGAGGAAAACTCCAGTAATTGCA<br>R: AAGTCGAAGAGGAAGGCCATAA	XM_046941902.1	(Su et al., 2014)
	SLC7A2	Solute Carrier Family 7 Member 2	Transport lysine. arginine. and histidine	F: TGCTCGCGTTCCCAAGA<br>R: GGCCCACAGTTCACCAACAG	XM_046916218.1	(Su et al., 2014)
	SLC7A5	Solute Carrier Family 7 Member 5	Transport hydrophobic amino acids	F: ACGTGCAAGCTCACACCTAA<br>R: CGAGGCCTCCTCAACTCTCA	NM_001030579.3	This study
	SLC7A6	Solute Carrier Family 7 Member 6	Na+-dependent neutral/cationic amino acid exchanger	F: GCCCTGTCAGTAAATCAGACAAGA<br>R: TTCAGTTGCATTGTGTTTTGGTT	XM_040681080.2	(Su et al., 2014)
	SLC7A7	Solute Carrier Family 7 Member 7	L amino acid transporter 2	F: CAGAAAACCTCAGAGCTCCCTTT<br>R: TGAGTACAGAGCCAGCGCAAT	XM_046911929.1	(Su et al., 2014)
	SLC2A1	Solute Carrier Family 2 Member 1	Glucose transporter-1	F: GCAAGATGACAGCTCGCCT<br>R: GCTCCTCATATCGGTACAGCC	NM_205209.2	This study
	SLC2A2	Solute Carrier Family 2 Member 2	Glucose transporter-2	F: CAGGAACGTTGGTCCTCTCC<br>R: GCGCCCATAGTGTGCTTCTA	NM_207178.2	This study
	SLC5A1	Solute Carrier Family 5 Member 1	Sodium glucose transporter 1	F: GCCATGGCCAGGGCTTA<br>R: CAATAACCTGATCTGTGCACCAGTA	XM_046928028.1	(Su et al., 2014)
	SLC2A5	Solute Carrier Family 2 Member 5	Transport fructose	F: AAAGAGCTGTAGGTGTGGGC<br>R: CTTTTGCCTGGTTGCCTTCC	XM_040689119.2	This study
	SLC5A9	Solute Carrier Family 5 Member 9	Sodium glucose transporter-4	F: ATACCCAAGGTCATAGTCCCAAAC<br>R: TGGGTCCCTGAACAAATGAAA	XM_020589419.5	(Su et al., 2014)
	FABP	Fatty Acid Binding Protein	Liver fatty acid binding protein	F: TGAATGTGGCTGGCTCGATTT<br>R: CAGGTTGACCCCTCCTGTACG	AY563636.1	This study
	FABP1	Fatty Acid Binding Protein 1	Fatty acid binding protein	F: CATCTTCTCTTGTGTTGGGAGC<br>R: TGATCATCAGGAAGCCCGAG	NM_204192.4	This study
	FABP2	Fatty Acid Binding Protein 2	Related with epithelial cell content and occurrence	F: ATGGAAGCAATGGGCGTGAA<br>R: TTCGATGTCGATGGTACGGA	NM_001007923.2	This study
	FABP6	Fatty Acid Binding Protein 6	Necessary for the transport of bile acids in the gut and it is associated with bacterial presence and inflammation	F: CGGTCTCCCTGCTGACAAGA<br>R: CCACCTCGGTGACTATTTTGC	XM_046926910.1	(Chen et al., 2016)
	SLC34A2	Solute Carrier Family 34 Member 2	Intestinal phosphate absorption and phosphate homeostasis	F: TGGGGAGAAAGAAGTGTCACAGA<br>R: GTGAAGCCACGTTGCCTTTGT	NM_204474.3	This study
	VDR	Vitamin D Receptor	Transcription factor that mediates the vitamin D3. involved in signalling intestinal calcium and phosphate absorption	F- GCAAAAGGCCGAGAAATGGG<br>R- GAACACCCGTGGCAGATTCA	XM_046934191.1	This study
	ATP1A1	ATPase Na+/K+ Transporting Subunit Alpha 1	ATPase Na+/K+ transporting subunit alpha 1 (Calcium transporter)	F- TGCAAATCCATCAGAATCTCGT<br>R- TCCTCATCCAAGGGTTGCAC	XM_046906976.1	This study
	SLC30A1	Solute Carrier Family 30 Member 1	Efflux of Zn2+	F: TGGGTGATATGAAGGAC<br>R: AACCTAAGGCATCTCCA	NM_001389457.2	This study
	CALB1	Calbindin 1	Calcium transporter	F- GGCAGGCTTGGACTTAACACC<br>R- GTCGGCAACACCTGAGCAAG	NM_205513.2	(Zanu et al., 2020)
**Metabolism**	*Cox-16*	Cytochrome c oxidase subunit 16	Enzyme involved in the generation of energy by the mitochondria	F: CCTGCTTTGAAGGAAAAATTGAAG<br>R: CCAAGTCAGATTGTTCCAATTTCTC	NM_001197057.2	(Criado-Mesas et al., 2021)
	EIF4EBP1	Eukaryotic Translation	mTOR pathway proteins—protein synthesis and cell proliferation	F: ATTGAGAACAACCATGTCCAGAAC<br>R: ATGTCAAACTGCTCTTCTTCACCT	XM_040689367.2	(Criado-Mesas et al., 2021)

		Initiation Factor 4E			
	mTOR	Mechanistic Target of Rapamycin	mTOR pathway proteins—protein synthesis and cell proliferation	F: TGCTGACAAACGCTATGGAGGT<br>R: AGCCATGACACTGTCCTTATGCT	XM_040689168.2	(Criado-Mesas et al., 2021)
	RPS6KB1	Ribosomal Protein S6 Kinase B1	mTOR pathway proteins—protein synthesis and cell proliferation	F: ACACCTGTTGATAGCCCAGATGA<br>R: GCCACATACGTAAAACCCAGAAA	XM_046930143.1	(Criado-Mesas et al., 2021)
**Oxidation**	*GPX7*	Glutathione Peroxidase 7	Intracellular antioxidant. and plays a great role in the detoxification of various peroxides	F: GGTGCCTCCTTTCCTATGTTCA<br>R: GTTGGTTCTTCTCCAGTAGAATCAA	NM_001163245.2	(Criado-Mesas et al., 2021)
	HIF1A	Hypoxia Inducible Factor 1 Alpha	Transcription factor that regulates genes involved in inflammation and cell death	F: CACTTTTTCAGGCAGTTGGAATTG<br>R: TTTTGCACGCCTTTACACGTT	XM_046917646.1	(Criado-Mesas et al., 2021)
	HMOX2	Heme Oxygenase 2	Oxidative stress marker	F: TCCAGTCCACGATGGGAAA<br>R: GCATTGCCTGCTAGCTTGTCT	XM_040684168.2	(Criado-Mesas et al., 2021)
	SOD1	Superoxide Dismutase 1	Antioxidant enzyme	F: CCGGCTTGTCTGATGGAGAT<br>R: CTGCGCTGGTACACCCATTT	NM_205064.2	(Criado-Mesas et al., 2021)
	XDH	Xanthine Dehydrogenase	Enzyme associated to the synthesis of reactive oxygen species and is member of cellular defense system	F: GAAGCCATTCCATTACTTCAGTTATG<br>R: AATGTCTGTGCGGATGTTCTTG	XM_046913189.1	(Criado-Mesas et al., 2021)
**Reference genes**	*LBR*	Lamin B Receptor	Reference gene	F: CTAACCGTCGCTCAGGGC<br>F: TCCAAAAGCAATACCTGGCG	NM_001396139.1	This study
	NDUFA	NADH Oxidoreductase Subunit A	Reference gene	F: TGTGCAGAAACTACAGGACAAACTG<br>R: AGGGAAAGCTCATTTTCAGCCT	NM_001097637.1	(Criado-Mesas et al., 2021)
	YWHAZ	Tyrosine 3-Monooxygenase/Tryptophan 5-Monooxygenase Activation Protein Zeta	Reference gene	F: GCAAGCAGAAAGCAAAGTTTTCT<br>R: TGTGATTGCTCCACAATCCCT	XM_046911632.1	(Criado-Mesas et al., 2021)
	GAPDH	Glyceraldehyde-3-Phosphate Dehydrogenase	Reference gene	F: CGTGCAGCAGGAACACTA<br>R: CAGATCGATGAAGGGATC	NM_204305.2	This study
	18S	18S Ribosomal RNA	Reference gene	F: ATTCCGATAACGAACGAGACT<br>R: GGACATCTAAGGGCATCACA	XR_006936397.1	(Chen et al., 2016)
	B-Actin	Beta-Actin	Reference gene	F: TGACTGACCGCGTTACT<br>R: GACCCACGATAGATGGGAA	NM_205518.2	This study
	UB	Ubiquitin	Reference gene	F: GGGATGCAGATCTTCGTGAAA<br>R: CTTGCCAGCAAAGATCAACCTT	X02650.1	(Barekatain et al., 2019)
	RPS7	Ribosomal Protein S7	Reference gene	F: GGCGCTGAGCGAGAAAGG<br>R: CTCCAGGAGAGCCTGGGATA	XM_040667252.2	This study
	B2M	Beta-2-Microglobulin	Reference gene	F: TACTCCGACATGTCCTTCAACG<br>R: TCAGAACTCGGGATCCCACTT	NM_001001750.4	(Barekatain et al., 2019)
	GUSB	Beta-Glucuronidase	Reference gene	F: GGCAGACTGGTCCTGTTGTTG<br>R: GGGTCCTGAGTGATGTCATTGA	NM_001039316.2	(Barekatain et al., 2019)
	TBP	TATA-Box Binding Protein	Reference gene	F: AGCTCTGGGATAGTGCCACAG<br>R: ATAATAACAGCAGCAAAACGCTTG	XM_046913188.1	(Barekatain et al., 2019)
	TUBAT	Tubulin Alpha	Reference gene	F: CAAGCATGAATGCCAACTCTCC<br>R: TCACGCATGGTTCGTCCT	NM_205444.2	This study
	r28s	28S Ribosomal RNA	Reference gene	F: GGCGAAGCCAGAGGAAACT<br>R: GACGACCGATTTGCACGTC	XR_006936395.1	(Barekatain et al., 2019)

32

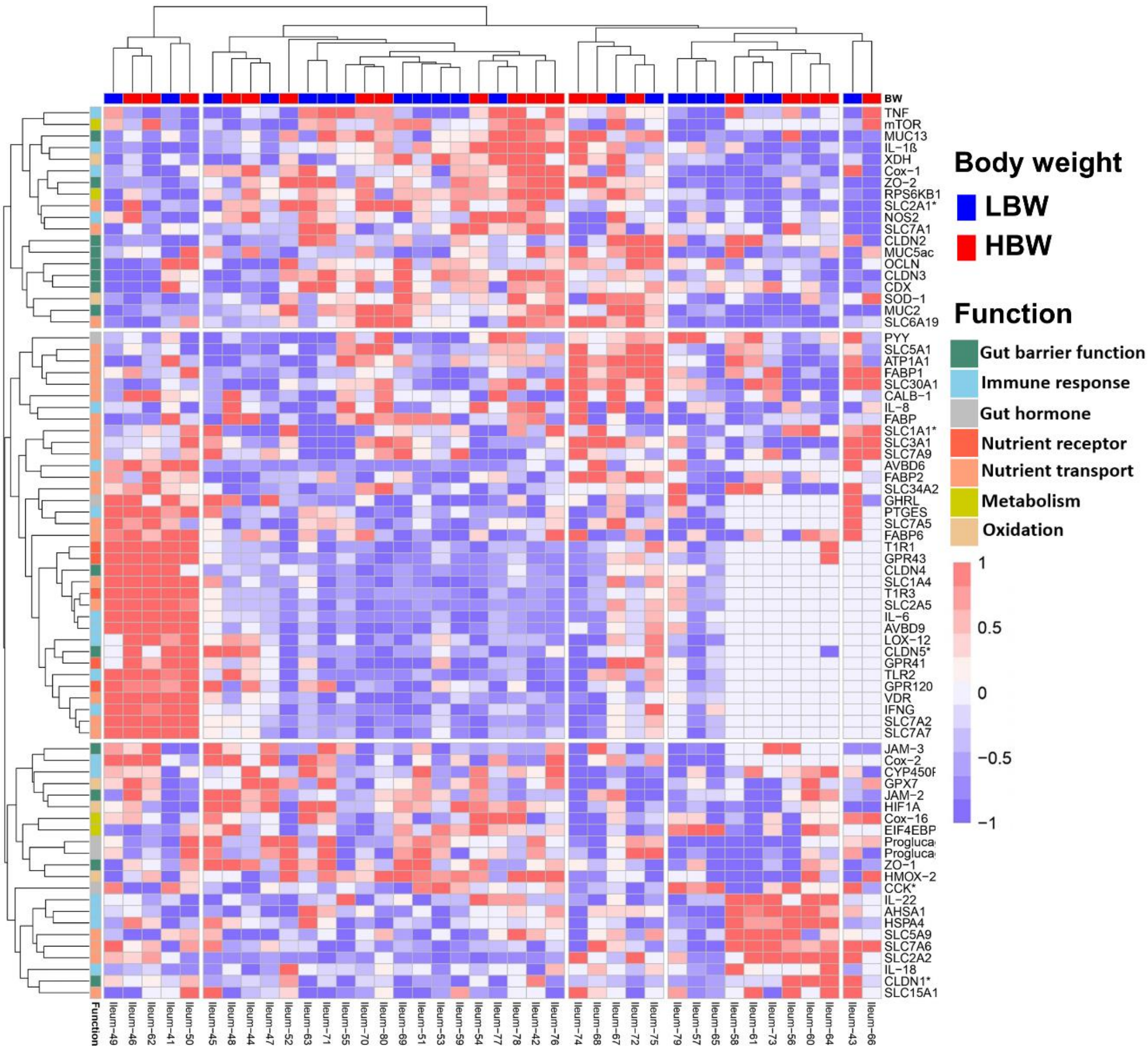

Fig. S1: Two-way hierarchical cluster analysis showing the expression level of the genes analyzed in the ileum between low (LBW, n = 20) and high (HBW, n = 20) body weight groups on day 14. Samples are represented on the x-axis and genes on the y-axis. The red color indicates high expression while blue indicates low expression. Gene functions (y-axis) were labeled with different colors. The dendrogram on the left of the heatmap clusters genes with similar expression patterns, while the dendrogram on the top groups samples with similar gene expression profiles. Genes with (*) indicate significant differences between BW groups based on the univariate analysis (Student's t-test).

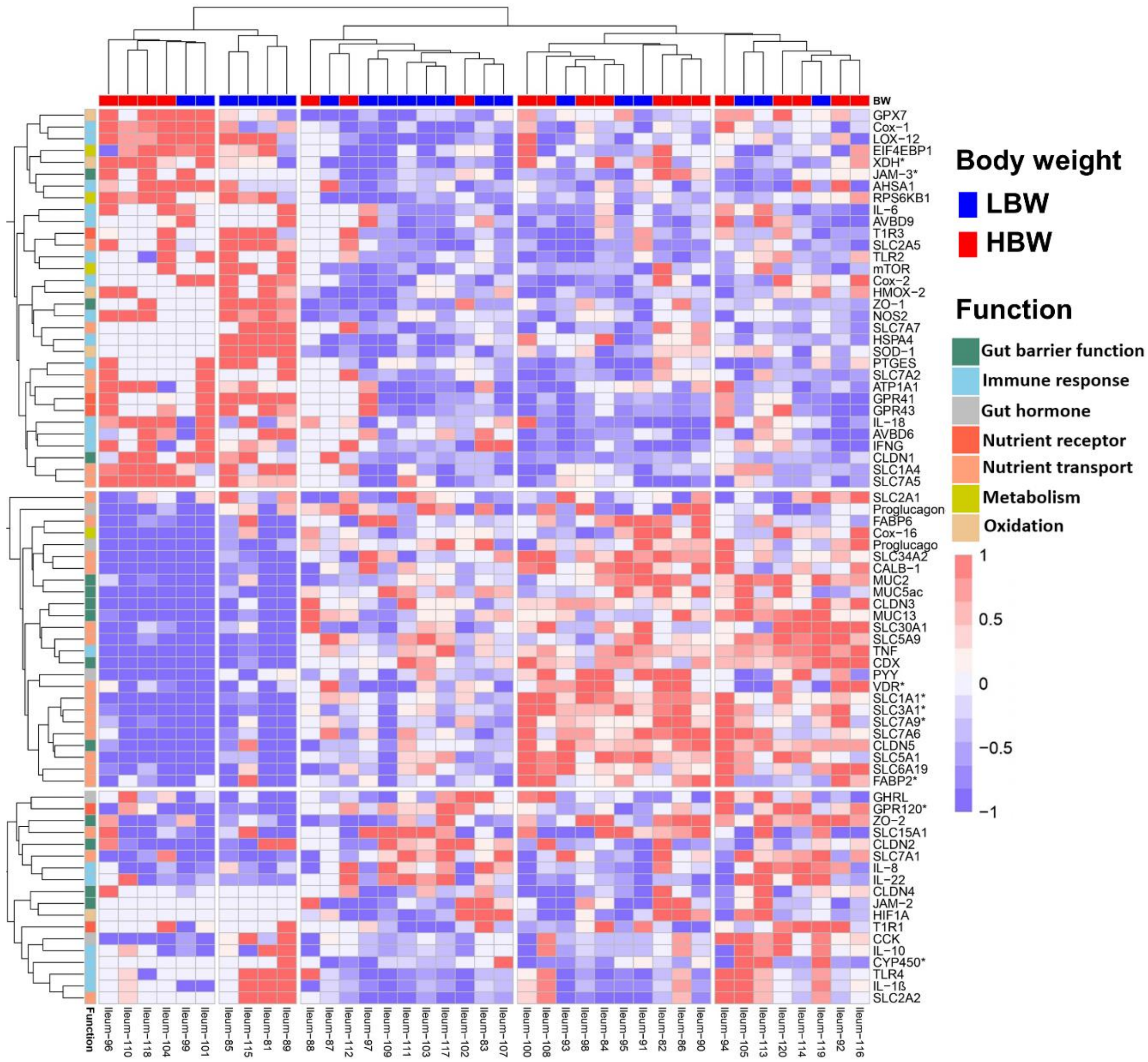

Fig. S2: Two-way hierarchical cluster analysis showing the expression level of the genes analyzed in the ileum between low (LBW, n = 20) and high (HBW, n = 19) body weight groups on day 38. Samples are represented on the x-axis and genes on the y-axis. The red color indicates high expression while blue indicates low expression. Gene functions (y-axis) were labeled with different colors. The dendrogram on the left of the heatmap clusters genes with similar expression patterns, while the dendrogram on the top groups samples with similar gene expression profiles. Genes with (*) indicate significant differences between BW groups based on the univariate analysis (Student's t-test).

References

Barekatain, R., P. V Chrystal, G. S. Howarth, C. J. McLaughlan, S. Gilani, and G. S. Nattrass. 2019. Performance, intestinal permeability, and gene expression of selected tight junction proteins in broiler chickens fed reduced protein diets supplemented with arginine, glutamine, and glycine subjected to a leaky gut model. Poult. Sci. 98:6761–6771.

Chen, X., K. Naehrer, and T. J. Applegate. 2016. Interactive effects of dietary protein concentration and aflatoxin B1 on performance, nutrient digestibility, and gut health in broiler chicks. Poult. Sci. 95:1312–1325.

Criado-Mesas, L., N. Abdelli, A. Noce, M. Farré, J. F. Pérez, D. Solà-Oriol, R. Martin-Venegas, A. Forouzandeh, F. González-Solé, and J. M. Folch. 2021. Transversal gene expression panel to evaluate intestinal health in broiler chickens in different challenging conditions. Sci. Rep. 11:1–14.

Forder, R. E. A., G. S. Nattrass, M. S. Geier, R. J. Hughes, and P. I. Hynd. 2012. Quantitative analyses of genes associated with mucin synthesis of broiler chickens with induced necrotic enteritis. Poult. Sci. 91:1335–1341.

He, X., Z. Lu, B. Ma, L. Zhang, J. Li, Y. Jiang, G. Zhou, and F. Gao. 2018. Effects of chronic heat exposure on growth performance, intestinal epithelial histology, appetite-related hormones and genes expression in broilers. J. Sci. Food Agric. 98:4471–4478.

Herwig, E., K. Schwean-Lardner, A. Van Kessel, R. K. Savary, and H. L. Classen. 2020. Assessing the effect of starch digestion characteristics on ileal brake activation in broiler chickens. PLoS One 15:1–20.

Hollemans, M. S., J. van Baal, G. de Vries Reilingh, B. Kemp, A. Lammers, and S. de Vries.

2020. Intestinal epithelium integrity after delayed onset of nutrition in broiler chickens. Poult. Sci. 99:6818–6827.

Konieczka, P., M. Barszcz, P. Kowalczyk, M. Szlis, and J. Jankowski. 2019. The potential of acetylsalicylic acid and vitamin e in modulating inflammatory cascades in chickens under lipopolysaccharide-induced inflammation. Vet. Res. 50:1–10.

Proszkowiec-Weglarz, M., L. L. Schreier, S. Kahl, K. B. Miska, B. Russell, and T. H. Elsasser. 2020. Effect of delayed feeding post-hatch on expression of tight junction– and gut barrier–related genes in the small intestine of broiler chickens during neonatal development. Poult. Sci. 99:4714–4729.

Song, Z., L. Liu, A. Sheikhahmadi, H. Jiao, and H. Lin. 2012. Effect of Heat Exposure on Gene Expression of Feed Intake Regulatory Peptides in Laying Hens (D Fan, Ed.). J. Biomed. Biotechnol. 2012:484869.

Su, S., K. B. Miska, R. H. Fetterer, M. C. Jenkins, and E. A. Wong. 2014. Expression of digestive enzymes and nutrient transporters in Eimeria acervulina-challenged layers and broilers. Poult. Sci. 93:1217–1226.

Zanu, H. K., S. K. Kheravii, N. K. Morgan, M. R. Bedford, and R. A. Swick. 2020. Interactive effect of dietary calcium and phytase on broilers challenged with subclinical necrotic enteritis: part 2. Gut permeability, phytate ester concentrations, jejunal gene expression, and intestinal morphology. Poult. Sci. 99:4914–4928.

Zhang, J. -M., X. -Y. Liu, W. Gu, H. -Y. Xu, H. -C. Jiao, J. -P. Zhao, X. -J. Wang, H. -F. Li, and H. Lin. 2021. Different effects of probiotics and antibiotics on the composition of microbiota, SCFAs concentrations and FFAR2/3 mRNA expression in broiler chickens. J. Appl. Microbiol. 131:913–924.

11

99
